# Semi-Empirical Calculation of Bodipy Aggregate Spectroscopic Properties through Direct Sampling of Configurational Ensembles

**DOI:** 10.3390/ijms231810955

**Published:** 2022-09-19

**Authors:** Sergey Usoltsev, Artem Shagurin, Yuriy Marfin

**Affiliations:** Department of inorganic chemistry, Ivanovo State University of Chemistry and Technology, 7 Sheremetevskiy Av., 153000 Ivanovo, Russia

**Keywords:** BODIPY, aggregate, ZINDO, XTB, sTDA, sTDDFT

## Abstract

Efficient prediction of the aggregation-induced callback of organic chromophores for utilization in molecular sensorics is a desirable development goal in modern computational chemistry. Dye aggregates are complicated to study when utilizing conventional quantum chemistry approaches, since they are usually composed of too many atoms to be effectively analyzed, even with high-throughput parallel systems. Here, we present a successful attempt to develop a protocol to assess the spectroscopic changes happening in BODIPY dyes upon aggregation from the first principles utilizing extended tight-binding (XTB) and Zerner’s intermediate neglect of differential overlap (ZINDO) Hamiltonians. The developed sampling technique for aggregate configurational space scanning was found to be sufficient to both reproduce peculiarities and justify experimental data on the spectroscopic behavior of chromophore aggregates. The sTDA, sTD-DFT (GFN2-XTB) and CIS (ZINDO) approaches were assessed, and then sources of errors and benefits were outlined. Importantly, our goal was to keep any of the mentioned calculations within a computational cost feasible for a single workstation, whereas scaling was possible at any point in time. Finally, several aggregate structures were investigated in the external field to try to achieve distributions similar to the ones observed in the electrostatic potential of the air–water interface to assess the borderlines of practical applicability of the suggested scheme.

## 1. Introduction

Dye aggregation is one of the challenges chemists face upon transitioning from flask investigations to complicated biological systems. Closed-order intermolecular interactions *in vivo* and in materials are known to drastically influence the spectroscopic properties. These interactions are currently lying in a gray area between gas phase and solid state investigations.

At this point of time, there are various protocols for the explanation of changes in the spectral properties of dyes due to solvent effects [1,2,3]. The photophysics of solid state systems, involving a far order treated by periodic boundary conditions, involve certain complications but are also currently calculated in a sophisticated manner [4,5,6]. However, “real” biological and technical systems are beyond these uniform, perfect conditions and involve multiple struggles, including dye aggregation.

Recent studies have shown pronounced and increasing interest in dye-containing functional materials accompanied by a systematic shift from utilization of single-molecule properties to molecular aggregate properties [7,8,9]. Dye aggregates are a complicated object of studies, since the potential energy surface even for two separate molecules in a dimer is quite noisy. The investigation of aggregates could require sampling more careful than that available from coarse-grained [10], molecular dynamics [11] or Monte-Carlo simulations [12].

Since there are very few if any available methods for thorough examination of molecular aggregates, somewhat simplistic approaches are often adopted. For example, an explanation of the tendency of a dye to exhibit aggregation-induced emission or aggregation-caused quenching is often sought from single-molecule intramolecular rotational barriers in the framework of the restriction of intramolecular motion and other theories [13,14,15]. Aggregate structures are assumed to be close to crystal structures for utilization in conventional calculations [16,17], and in general, when it comes to description of spectroscopic evidence, either phenomenological theories or single-molecule properties are mostly utilized for their analysis [18,19].

There is also an important complication in computational approaches to dye investigations. Usually, aggregate bands are described in experimental research in a quite sparse manner unless they are nice, separate bands. Validation of the calculation results thus becomes a significantly tedious task. Two important counterexamples in boron-dipyrrin (BODIPY) chemistry are articles by Descalzo et al. [20] and Kim et al. [21], which accumulated very careful examinations of changes happening in selected groups of BODIPY compounds and provided design considerations of J-aggregate-forming molecules.

However, even in this case, conclusions are still usually based on single-molecule parameters, even though the synergetic nature of aggregation-induced spectroscopic effects is evident. In our previous research, we also faced a need for explaining a certain processes happening in unideal solutions for BODIPY dyes. The latter were demonstrating structure-related peculiarities in absorption and emission, as shown in [22,23].

Highly sophisticated examinations of electronic excitations are available from multireference (MR) methods, such as complete active space second-order perturbation theory (CASPT2) or n-electron valence state second-order perturbation theory (NEVPT2) [24,25]. It’s quite common to use such multi-reference methods to benchmark lower-level approaches. However, there are troubles concerning system size; the mentioned methods are extremely computationally demanding (no more than ∼20 orbitals can be correlated) [26,27,28]. They also require a justified choice of active space for each specific case.

These methods, unluckily, were never benchmarked for utilization with big molecular aggregates. Ultimately, MR methods serve the purpose of describing the electronic structure exceptionally well, but their applicability to BODIPY aggregate systems is questionable.

Time-dependent density functional theory (TDDFT) methods are among the most utilized approaches for the explanation of geometry and excited state properties. They are generally considered a best standard in cost-efficiency and applicability for proper explanation of the electronic properties of chromophores, especially in terms of shifts [29,30,31]. Unluckily, even with smart simplifications of TDDFT and widely co-utilized Tamm–Dancoff approximation (TDA), size limitations emerge at the point of time the aggregates are in the spotlight.

The best alternative candidates for the explanation of aggregate systems are regaining attention: semi-empirical quantum chemistry (SEQC) methods [32].

The recently introduced extended tight binding (XTB) approach is showing rapid expansion into various fields of calculations, which was previously unfeasible by most of the methods. XTB successfully deals with large dispersion-interaction bound supramolecular complexes and biomolecules [33,34].

Along with tight-binding methods, there are simplified the Tamm–Danchoff approach (sTDA) [35] and simplified time-dependent density functional theory (sTD-DFT) [36] methods for calculation of the excited state properties. These methods allow for highly accurate predictions of the vertical excitation energies of large systems utilizing XTB Hamiltonian.

Zerner’s group formulation of intermediate neglect of diatomic overlap (ZINDO/S) [37,38] served as the only means for calculation of the spectral properties of large systems for decades [39,40]. Even though this method possesses certain crucial approximations, which are discussed in detail in Supplementary Materials Section S1, up to now, this has been a method of choice with a reasonable trade-off between speed and precision. It was also shown that the ZINDO/S method reproduces the vertical transition energies of the Frenkel and CT states reasonably well [41,42].

In the beginning of the 21st century, it was believed that increasing the computational throughput of future clusters would make any calculations accessible, but current trends show that the situation is not that trivial, and current research serves the purpose of demonstrating how simple instruments can yield valuable information upon a careful approach.

## 2. Materials and Methods

### Aggregate Sampling

**Figure 1:**
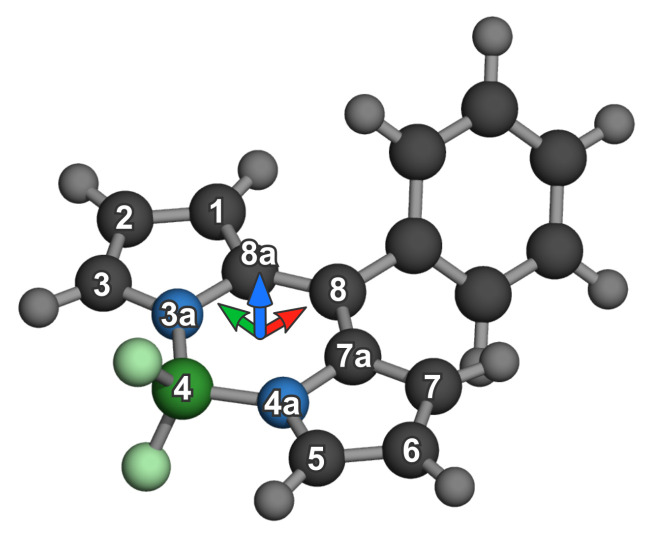
Generic BODIPY molecule with labels from indacene notation and axes as oriented prior to aggregate generation (blue for *z*-axis, red for *x*-axis and green arrow for *y*-axis).

Investigation of an aggregate configurational ensemble was performed by direct generation of a set of supermolecules. The atom coordinate matrix was iteratively transformed Equation (1) to obtain an n-size stack with various step amounts and sizes, discarding all of the conformations with overlapping atoms (lying within less than 1.5 from each other). Each configuration was also rotated 180° to account for the possibility of the formation of an antisymmetric pattern:

(1) $$\left[\begin{array}{c} x_{i}^{\prime }\\ y_{i}^{\prime }\\ z_{i}^{\prime }\\ 1 \end{array}\right]=\left[\begin{array}{cccc} cos\;\theta & -sin\;\theta & 0 & t_{x}\\ sin\;\theta & cos\;\theta & 0 & t_{y}\\ 0 & 0 & 1 & t_{z}\\ 0 & 0 & 0 & 1 \end{array}\right]\left[\begin{array}{c} x_{i}\\ y_{i}\\ z_{i}\\ 1 \end{array}\right]$$

where θ is the rotation angle around the z coordinate (orthogonally facing the BODIPY plane and going trough the centroid of **4**, **7a**, **8a** atoms in indacene notation (Figure 1)), $t_{n}$ is the displacement and n=x,y,z stands for the corresponding components of the translation vector.

**Figure 2:**
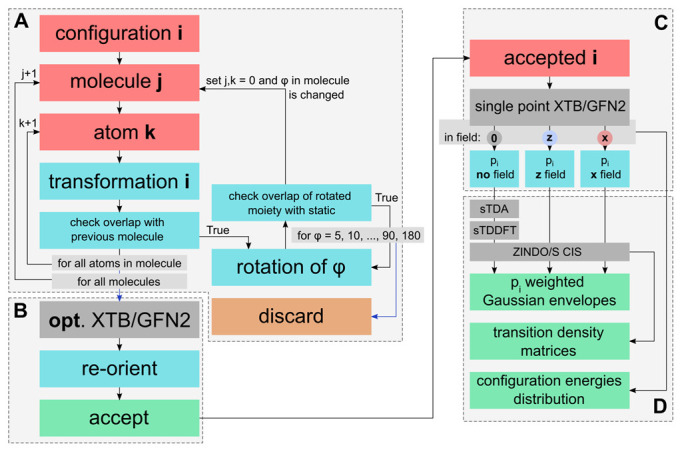
Block diagram of a calculation procedure. Dashed line blocks denote (**A**) sampling, (**B**) orientation, (**C**) ensemble analysis and (**D**) property calculation. Red blocks indicate input data, teal blocks indicate manipulations, gray blocks indicating external calculations, and green blocks indicate output data. Light gray blocks are cyclic processes, with blue showing the exit direction.

Atoms were placed one by one, and each subsequent placed atom was checked for overlapping ($d_{AB}<1.5$) with the original molecule according to the distance formula (Equaiton (2)). If this was the case, then either structure was modified to accommodate transformation. If any applied modification was unsuccessful, then the structure was discarded (Figure 2A):

(2) $$d_{AB}=\sqrt{\sum_{c}{\left(c_{B}-c_{A}\right)}^{2}},\;c=\left\{x,y,z\right\}$$

Here, structure modification need was dictated by the fact that occasionally, the geometrical topology was complicated, and the procedure sometimes yielded too small an amount of configurations. Prior to sampling, we chose the dihedral angle that influenced the structural complexity the most (the angle between the BODIPY moiety and the μ-substituent). As stated before, if during generation an atomic overlap happened, this moiety was rotated around the dihedral in 5° increments from −90 to 90° and 180°. During this rotation, we were also checking for self-overlap of the substituent and main moiety. At this point in time, intermolecular overlap was not happening anymore, and the corresponding rotation angle was used for further optimization.

This approach allowed us to increase the number of accepted initial configurations up to three times for the BODIPY structures with bulky aromatic substituents discussed later. Rotation was performed according to the Rodrigues procedure, which was convenient to use in a vector notation (Equation (3)):

(3) $$v^{\prime }=v\cdot cos\;\phi +\left(k\times v\right)sin\;\phi +k\cdot \left(k\cdot v\right)\left(1-cos\;\phi \right)$$

where *v* is an original vector, *k* is a unit vector defining the rotation axis, ϕ is a rotation angle according to the right-hand rule, and finally, $v^{\prime }$ denotes the rotated vector.

Each configuration was optimized in a GFN2-XTB level of theory, reoriented, and the coordinates were written to a file to form an xyz trajectory (Figure 2B).

Reorientation was performed according to the following procedure (Equation (4)):

(4) $$\begin{array}{cccc} \; & 1.\; & C_{n} & =\left[\sum_{ABCD}X_{i}\right]/4\;C_{01}=\left(C_{0}+C_{1}\right)/2\\ \; & 2.\; & X_{i}^{\prime } & =X_{i}-C_{01}\\ \; & 3.\; & C_{n} & =\ldots (1)\\ \; & 4.\; & i & =\left(A-A_{1}\right)\times \left(B_{1}-A_{1}\right)-C_{01}\\ \; & \; & j & =C_{0}-C_{01}\\ \; & \; & k & =i\times j\\ \; & 5. & i^{\prime } & =i\\ \; & \; & j^{\prime } & =j-\frac{i^{\prime }\cdot j}{i^{\prime }\cdot i^{\prime }}\cdot i^{\prime }\\ \; & \; & k^{\prime } & =k-\frac{i^{\prime }\cdot k}{i^{\prime }\cdot i^{\prime }}\cdot i^{\prime }-\frac{j^{\prime }\cdot k}{j^{\prime }\cdot j^{\prime }}\cdot j^{\prime }\\ \; & 6. & i^{\prime } & =\frac{i}{{\left||i|\right|}_{3}},\;j^{\prime }=\frac{j}{{\left||j|\right|}_{3}},\;k^{\prime }=\frac{k}{{\left||k|\right|}_{3}}\\ \; & 7. & X_{i}^{\prime } & =B\cdot X_{i}\\ \; & 8. & C_{tot} & =\left[\sum_{i}X_{i}\right]/i\;X_{i}^{\prime }=X_{i}-C_{tot} \end{array}$$

At the first step, the centroids of A,B,C and *D* and $A_{1},B_{1},C_{1}$ and $D_{1}$, denoted as $C_{0}$ and $C_{1}$ respectively, were calculated. Points A,B,C and *D* were 1-, 3-, 5- and 7-position carbons of BODIPY in indacene notation, and letters with an underscore index 1 denote the same exact atoms on the last odd BODIPY molecule in the aggregate. Using a last odd molecule was required since 180° alteration would otherwise affect the alignment process.

The centroid of points $C_{0}$ and $C_{1}$, named $C_{01}$, was then (in step 2) set as the center of a coordinate system for further calculations. The third step was reevaluation of the centroids from step 1 according to the new origin. Afterward, the basis vectors for alignment were generated so that the *y* axis (vector *j*) was oriented facing from $C_{01}$ toward $C_{1}$, the *x* axis was facing to the side $A,A^{\prime },B^{\prime },B$ of a prism, and finally, their cross-product was vector *j*.

**Figure 3:**
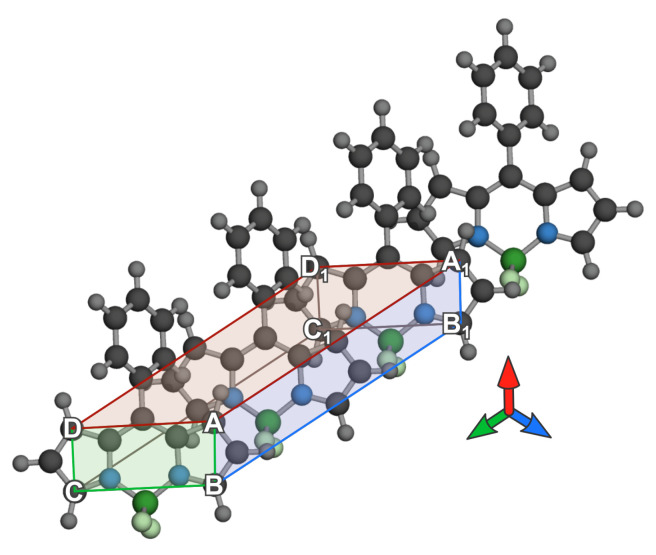
Oriented aggregate of BODIPY molecules with indicated axes orientations (blue for *z*-axis, red for *x*-axis orthogonal to ABB${}_{1}$A${}_{1}$ and green arrow for *y*-axis orthogonal to ABCD).

Since the ABCDA’B’C’D’ prism was skewed for almost all of the configurations, vectors i,j and *k* were additionally orthogonalized via the Gram–Schmidt process (step 5) to avoid deformations upon a change in basis. All of the vectors were then normalized in step 6. The basis was then applied to each point $X_{i}$, and since our centroid at the time did not correspond to a global centroid if the number of molecules in the aggregate was odd, a new global centroid was calculated and applied as the origin in the last step (example results in Figure 3).

Careful reorientation was required to grade the configurations according to their energy in an anisotropic electric field to assess the relative probabilities of these configurations as if they were oriented as “flat” or “standing” in the air–water interface potential. Similarly, the orientation was required for calculations of the vertical transitions in the same conditions.

The probabilities of the corresponding configurations both with and without an external field were evaluated from a Boltzmann distribution according to their GFN2-XTB electronic energies (Figure 2C):

(5) $$p_{x}=\frac{exp\left[\frac{-(e_{x}-e_{0})}{kT}\right]}{\sum_{i}exp\left[\frac{-(e_{i}-e_{0})}{kT}\right]}$$

with the ensemble temperature *T* selected as 293.15 and Boltzmann’s constant $k_{B}=3.1668\times 10^{-6}$ E${}_{h}\cdot$K${}^{-1}$.

Only configurations with a sufficient relative probability ($p_{x}>0.02$) were taken for the further calculations. The vertical electronic excitations recovered from the sTDA, sTDDFT and ZINDO/S-CIS calculations were broadened with a Gaussian shape with an arbitrarily chosen 15-nm variance (so that the bands were wide enough to visually resemble the absorption spectrum but were not too narrow to overcrowd it upon envelope generation) and scaled linearly to their corresponding oscillator strengths (Equation (6)):

(6) $$A_{i}\left(\lambda \right)=\frac{f_{osc}}{\sigma \sqrt{2\pi }}\cdot exp\left[\frac{-{\left(\lambda -\mu \right)}^{2}}{2\sigma^{2}}\right]$$

All of the envelopes were then added together after rescaling according to their relative probability to collect an ensemble vertical absorption envelope for each molecule (Figure 2D).

For the reader to get a broad image of the sampling and calculations, Figure 2 summarizes the protocol in a visual block diagram.

All of the calculations were performed for the whole range of 14 molecules introduced in the following section.

The calculations were automated with custom Python scripts, utilizing the NumPy [43] and SciPy [44] libraries, and graphs were plotted utilizing the matplotlib [45] package. Structures were visualized with the PyMol package [46]. The XTB release 6.4.1 [47] and DFTB+ release 22.1 [48] packages were utilized for molecular structure optimizations and for single-point calculations in an anisotropic electric field. Orca release 5.0.2 [49] was utilized for the ZINDO/CIS calculations in Tamm–Dancoff approximation. Multiwfn version 3.8 [50,51] was utilized for Hirschfield surface analysis [52,53] and Hirschfield fingerprint construction. Multiwfn was also utilized for fragment transition matrix construction based on a transition density matrix (TDM) [54,55,56] without symmetrization. Fragment-partitioned matrices were calculated as the square roots of the sum of the square of TDM elements (as implemented in Multiwfn).

## 3. Results and Discussion

### 3.1. Rational Sampling

Configurational ensembles of aggregates were generated via brute-forcing possible molecular co-orientations in order to assess a noisy intramolecular potential energy surface for a one-dimensional periodicity (linear) system.

The molecule for assessment, 8-phenyl BODIPY (named hereafter BODIPY for convenience), was chosen for being one of the most generic possible structures of its kind.

It was important to evaluate which amount of molecules in the bunch and which step amount were sufficient for further examinations. There were two considerations: electronic energy distribution of the sampled supermolecules represented by a configuration probability distribution (CPD) histogram and the impact of changes in the sampling procedure on the resulting spectral envelopes.

The dimers were not evaluated in this article, as they should rather be considered non-periodic systems and have already been thoroughly studied. All of the aggregates constituted at least three BODIPY molecules (but all the findings can also easily be generalized to dimers).

The maximum displacement was chosen to be six in order to avoid bunch disengagement, as such a configuration would span further into the area of 2D and 3D aggregates. The variable in the process of procedure assessment was the amount of steps between the minimal and maximal displacements.

Configurations on the *x* and *y* axes came in (4n+1) successions each, including zero displacement and 2n steps in each direction on the corresponding axis. The total amount of displacements on the *z* axis (interplanar distance) was (n+1), since no negative displacement was considered. The amount of rotation steps was always 2 (0° and 180°).

The total amount of configurations generated prior to discarding the overlapping ones was thus their product, $2\cdot \left(n+1\right)\cdot {\left(4n+1\right)}^{2}$, leaving us with total of 100 configurations for *n* = 1, 486, for *n* = 2, 1352, for *n* = 3 and so on (*n* is referred to in the following text as a generation process step amount).

**Figure 4:**
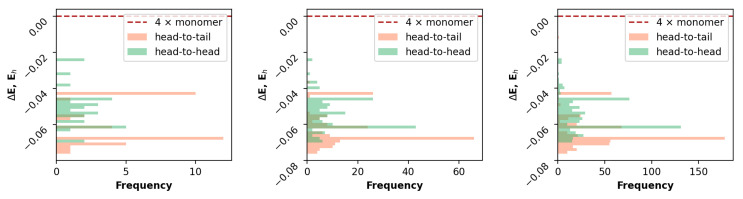
Distribution of GFN2-XTB electronic energies of sampled aggregate configurations for 4 molecules of BODIPY for 1–3 steps of the generation process (left to right).

As can clearly be seen from the example CPD for the BODIPY aggregates of four molecules (Figure 4), the changes became insignificant at the second step. Note that head-to-head and head-to-tail classification were utilized and not a commonly found H and J aggregate notation. This is important, because the latter is devoted to a mutual orientation of transition dipoles in exciton theory [18] or consecutive spectroscopic changes (depending on the context), whereas the former is solely a geometrical designation obtained from initial mutual rotation of consecutive molecules in an aggregate.

Generally, the step amount could be increased to smooth out the distribution, and in the future, we will come up with a process to discard close optimized structures, but since at this point in time one of our major goals was to keep the calculations within a single-machine workload, 486 total configurations yielded by the two-step process was considered sufficient.

Spectral changes upon a step amount increase (Figures S1–S6) were found to be insignificant at the same point and only demonstrated changes in intensity (weighted oscillator strengths). Despite that fact, an *a posteriori* check was performed for all of the calculations mentioned in the article to ensure ensemble representativity for all of the performed calculations.

**Figure 5:**
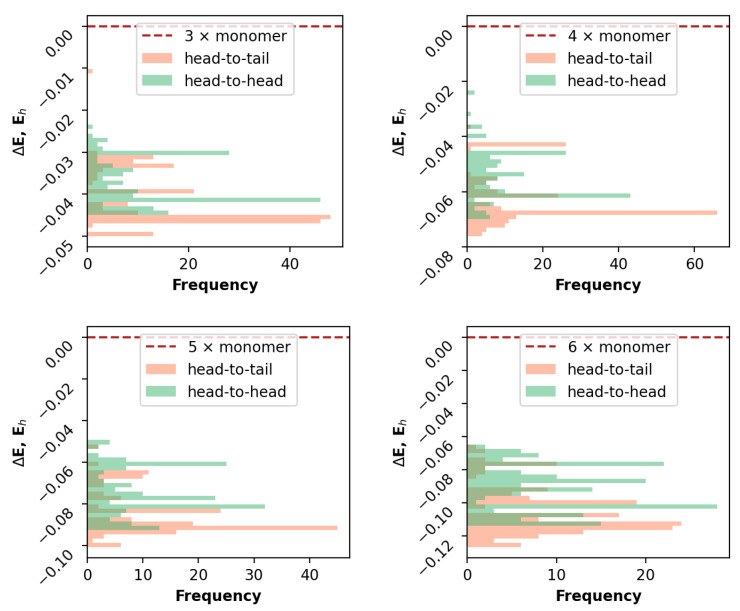
Distribution of GFN2-XTB electronic energies of sampled aggregate configurations for different amounts (3–6) of molecules of BODIPY generated from the two-step process.

The amount of molecules in the aggregate was quite a controversial variable in the examination. As can be seen from the CPD in Figure 5, the distributions were not altered significantly and were preserved in almost exactly the same shape and relative abundances. Remarkably, the relative energies of the aggregates systematically decreased upon an increase in the molecule amount.

This effect should probably highly depend on the kind of molecule upon examination and could, to some extent, help draw a line between cooperative and isodesmic aggregation processes.

We preserved the details for our ongoing research, and since here we emphasize the spectral properties of aggregates, it is sufficient to mention that the band shapes did not alter significantly upon an aggregate size increase. The exception was when the brightest transition was increasingly surrounded by bands with almost vanishing oscillator strengths devoted to localized excitations (the bands are investigated in detail via transition density matrices in further sections). The positions of these bands were dictated by differences in the environment of each and every molecule, thus being an artifact of a gas phase calculation, potentially compromising the result quality and significantly slowing down calculations. In the sTDA and sTD-DFT calculations, this kind of crowding led to visible redistribution of the band intensities Figures S1–S6), indicating that these methods are more susceptible to long-range interactions during excitations, which is not necessarily a beneficial behavior yet has to be kept in mind in further examinations.

With all of the above-mentioned caveats, we stated four molecules to be a sufficient aggregate size.

**Figure 6:**
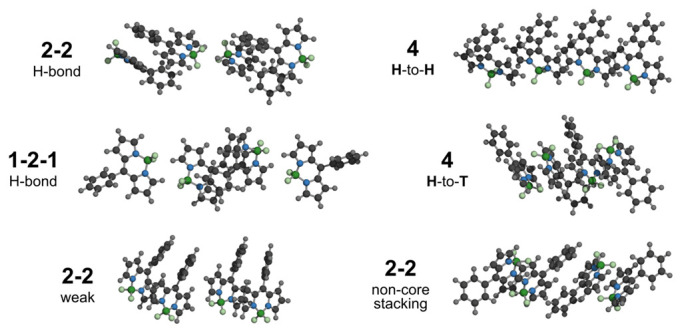
Examples of optimized configurations of four BODIPY structures with peculiarities in intermolecular interactions (2-2 and 1-2-1), along with examples of close-ordered tetrameric structures with head-to-head (4 H-to-H) and head-to-tail orientations (4 H-to-T).

It is worth noticing that due to the optimization step, our results lack predefined aggregate geometry assumptions, so we also assessed the possibility of the formation of two dimers, or 1-2-1 configurations, instead of total stacking. Examples of the interaction peculiarities are shown in Figure 6 along with the usual tetramers for visual comparison. It could happen that for some molecular structures, such configurations become favorable, and thus examining aggregates involving less than four molecules could lead to calculating erroneous results.

### 3.2. Comparative Analysis

The molecules for examination were selected from multiple sources, including molecules investigated and under investigation by our group. The approach to labeling as well as the set of generic structures was taken from Descalzo et.al. [20], and “R” at the beginning of the molecule name was used to point to the number of BODIPY core substituents such that R0 stood for a fully unsubstituted core, R2 stood for the α-dimethyl derivative, R4 was the $\alpha ,\alpha^{\prime }$-tetramethyl derivative and R6 was the β-diethyl-$\alpha ,\alpha^{\prime }$-tetramethyl derivative, as produced from common commercially available pyrroles.

The molecule previously called BODIPY was named **R0 Ph** in this row.

Ph, Naph, Anht and Pyr are a set of μ-substituents with a varying number of conjugated rings, corresponding to μ-phenyl, μ-naphtyl, μ-anhthryl and μ-pyrenyl, the substituted BODIPY dyes.

From the phenyl-substituted dye, the genetic relatives are the p-dimethylammino and p-dibuty-lammino derivatives along with the p-decyloxy and p-octadecyloxy derivatives.

**Figure 7:**
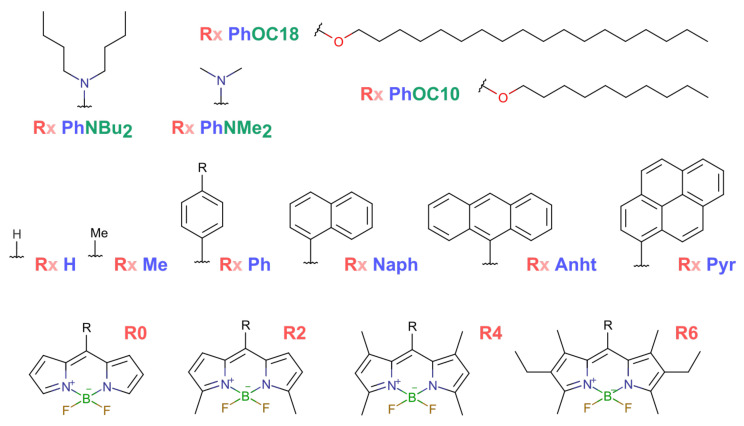
Structures of investigated compounds. In Rx, x stands for amount of alkyl chain substituents in the BODIPY core (found in the bottom row), the middle row shows varying meso-substituents, and the top row depicts various groups in the ortho-position of the phenyl moiety.

In total, we came up with a set of 14 molecules with quite distinct organizational and photophysical properties (Figure 7).

According to the substitution pattern, the molecules were divided into four major (connected) groups as follows. The R0–R4 bearing compounds did not possess the β-ethyl moiety stated in the literature as being responsible for a decreased aggregation tendency, combined into group **(I)**.

Groups **(II)**, **(III)** and **(IV)** fully consisted of BODIPY dyes with dense alkyl substitution (R6) in a chromophore unit. Group **(II)** consisted of dyes with various size aromatic moieties in a μ-position and one dye without any substituent, and groups **(III)** and **(IV)** were μ-phenyl dyes with nitrogen- and oxygen-bearing para-substituents corresponding with varying alkyl chain lengths.

The compound classification according to their structural features is summarized below:

 **(I)**  *Sparse core substitution*: **R0 Ph**, **R2 Ph**, **R4 Ph**, **R4 Me** and **R4 H**;
 **(II)**  *Different size aromatic moiety:* **R6 H**, **R6 Ph**, **R6 Naph**, **R6 Anht** and **R6 Pyr**;
 **(III)**  *Donating nitrogen in μ-position:* **R6 PhNMe${}_{2}$** and **R6 PhNBu${}_{2}$**;
 **(IV)**  *Donating oxygen in μ-position and long aliphatic:* **R6 PhOC10** and **R6 PhOC18**.

#### 3.2.1. Geometrical Organization

Depending on the steric complexity of a single molecule, the sampling process yielded different total amounts of configurations for consideration. Nonetheless, even the smallest amount (a total of 17 configurations) obtained for **R6 Anht** was still above the amount of configurations usually considered in similar investigations. The compounds in series **(I)** had the lowest-lying head-to-tail configurations (with a low twist or offset, except for **R0 Ph**). There were some differences in the exact spatial parameters, yet they were not obvious and are discussed in detail later.

CPD histograms for all of the compounds are presented in Figure S7.

In series **(II)**, only **R6 H** had the highest probability of head-to-tail organization, but due to the steric hindrance imposed by the β-ethyl groups, a vast ensemble of poorly organized molecules was obtained after optimization. The rest of the group, consisting of compounds with different amounts of condensed rings in the μ-position, had the most probable head-to-head organization. Compared with series **(I)**, these aggregates had larger intermolecular distances with vacant spaces between the aromatic substituents, suggesting that series **(II)** could form more organized 2D or 3D aggregates. Apart from the lowest energy configuration, there was sensible ambiguity in the type of aggregate formed, except for **R6 Naph**, the ensemble for which was strongly dominated by head-to-tail configurations.

The compounds in series **(III)** both had the lowest-lying head-to-tail configuration, but for **R6 PhNMe${}_{2}$**, ambiguity was present, as can be seen in the CPD histogram.

In turn, the aggregates formed by the compounds in series **(IV)** were mostly of the head-to-head type, probably due to the strong effect of a long alkyl chain.

**Figure 8:**
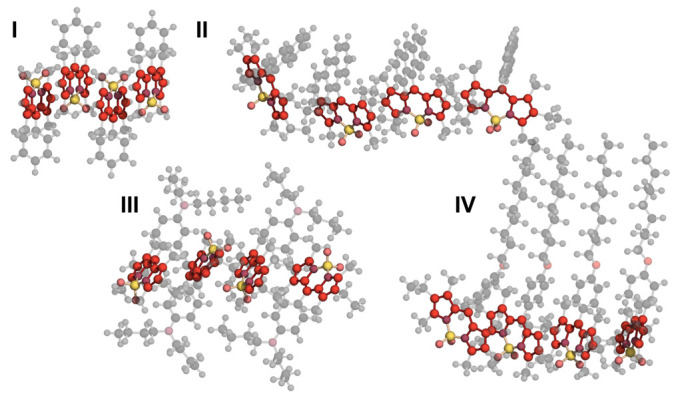
Aggregate structures common for groups of compounds and lowest-lying configurations: I. **R4 Ph**, II. **R6 Anht**, III. **R6 PhNBu${}_{2}$** and IV. **R6 PhC10**.

Information about generated configuration ensembles is presented in (Table 1), common structures of lowest lying aggregate configurations representative for compounds in groups **(I–IV)** could be seen in Figure 8.

The lowest energy aggregate structures for all of the compounds for visual inspection are presented in Figures S8 and S9.

A deeper investigation of the organizational properties was performed utilizing Hirschfield surface (fingerprint) analysis. Though this method was originally developed for investigating crystal structures, we were able to obtain even more information about the aggregates because the environments of the different molecules in the aggregates were varied to the extent of the overall organization.

**Figure 9:**
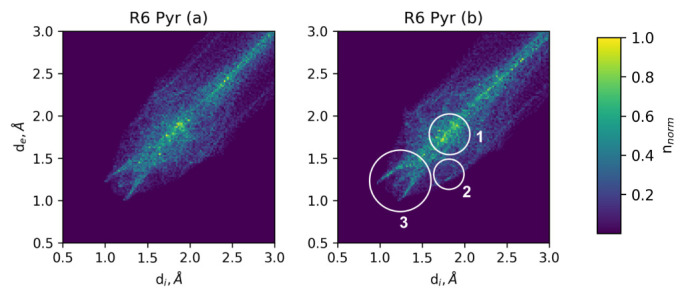
Example of Hirschfield fingerprint analysis plane for **R6 Pyr** with visibly asymmetric off-diagonal interaction modes (different patterns in histograms (**a**,**b**)). Circled regions of interest are (**1**) peak corresponding to planar stacking, (**2**) asymmetric interaction modes (mostly C–H in this case) and (**3**) cyclic H-bonding pattern of H-F bonds.

Similarity of the fingerprints means high organization inside the aggregates, whereas large dissimilarity obviously points to differences in the interaction modes. Partitioning of the Hirschfield surfaces according to the pairs of atoms involved in an interaction also shed light on the reasons for tight or sparse aggregation. An example of fingerprints with partitions can be seen at Figure 9, and fingerprints along with atom pair partitioning are presented in Figures S12–S15.

**Figure 10:**
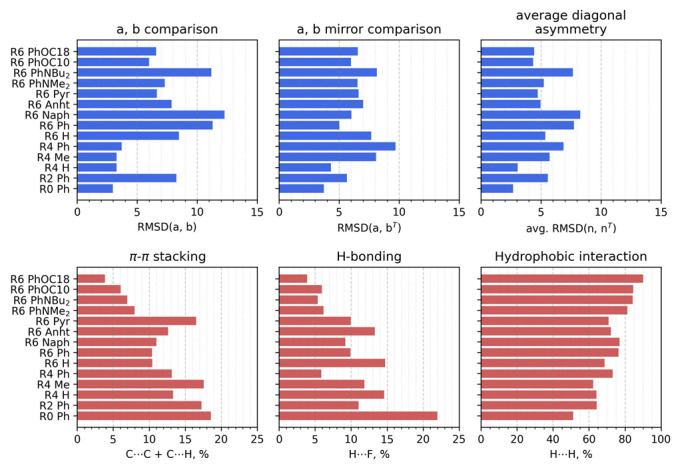
Comparative analysis of Hirschfield fingerprints for BODIPY aggregates. Letters a and b denote two (non-terminal) different molecules inside the tetramer.

For quantitative analysis, we utilized several characteristics. First, we used the root mean square deviations (RMSDs) of the corresponding d${}_{i}$ and d${}_{e}$ from the fingerprint diagrams of different molecules for similarity evaluation. Second, we included the d${}_{i}$ and d${}_{e}$ values of one molecule with a transposed histogram of another molecule to evaluate mirror similarity. Finally, each fingerprint was divided onto a lower and upper triangle, and the latter were then also compared to evaluate the symmetry of the interaction modes within each single molecule (Figure 10).

Intense spots in the fingerprints are owing to highly planar stacking interactions and mostly corresponding to C–H and C–C atom pairs. Off-diagonal spikes correspond to several polarized spots on a Hirschfield surface, such as H–F (and F–H) pairs, as well as some small inputs of other interactions.

Upon rational simplification, we could say that the C–C and C–H inputs for the Hirschfield surface belonged, in our case, to stacking interactions, and the H-F inputs could be considered hydrogen-bonding interactions (due to the largely off-diagonal manner of the corresponding pairs on the fingerprint plots and related critical points), whereas the H-H inputs were mainly responsible for hydrophobic interactions. This simplification was only reasonable for the sake of quantitative comparison of the fingerprints, and usually, direct visual inspection is preferred.

Only the compounds in series **(I)** were characterized by the high similarity of Hirschfield fingerprints within the aggregates, except for the compound **R2 Ph**. The highest dissimilarities were observed for **R6 PhNBu${}_{2}$**, **R6 Ph** and **R6 Naph**. In this case, the lack of perfect regularities should be noted, meaning some structural modifications affect organization to a larger extent, and some are more subtle and thus should not be generalized. One similar modification motif, the change in a length of aliphatic moiety, influenced the difference between **R6 PhNBu${}_{2}$** and **R6 PhNMe${}_{2}$** to a larger extent than for **R6 PhOC10** and **R6 PhOC18**.

In most of the structures for which the RMSD between molecules was high, the RMSD with the mirror image of a fingerprint was low, meaning there were varying tendencies for partitioning onto two dimers instead of tetramer formation. A bright and important example of this behavior was **R2 Ph**, for which interaction between the inside molecules was realized through stacking between the BODIPY chromophore moiety of one molecule and the phenyl moiety of another. We thus conclude that there was a high importance of such a comparison for a fast and efficient examination process.

The diagonal symmetry of a fingerprint in turn describes how aligned interactions are within trimers, and the qualitative pattern was similar to the mirrored pair RMSD. The exception was the outlying **R6 Ph** and **R6 Naph** aggregates with high asymmetry, but since they were also different within the pairs, in this case, we were only able to state an overall high disorder in the aggregates of these compounds.

Partitioning onto interaction pairs shed light on the observed discrepancies. In general, an increase in the amount of aliphatic substituents and their lengths led to a logical increase in the normalized input of H–H pairs onto the Hirschfield surface. Though the main counterparts did not change monotonically, **R6 Pyr** and **R6 Anht** showed significant input from the stacking interactions (more pronounced for the former) and H-bonding (for the latter). This pointed out that these interactions tend to be responsible for higher structuring of the aggregates of these molecules. Within series **(I)**, the input of stacking for BODIPY **R2 Ph** was also large, but since the stacking was misaligned, in this case, it did not serve the purpose of organization.

As for mutual orientation of the molecules, compounds with intense stacking interactions usually aligned in a head-to-tail orientation, but different forces were responsible for the opposite. In the case of series **(II)**, the H-F input dominated, and in the case of series **(IV)**, hydrophobic interactions drove the head-to-head orientation.

#### 3.2.2. Field Susceptibility

It was particularly interesting to switch from investigations of the aggregates *in vacuo* to structures in a model confinement. After orientation, the molecular aggregates were subjected not just to grading according to their electronic energy but also according to their single-point energy in an anisotropic electric field in an attempt to mimic the influence of the interfacial potential on the aggregate geometry. The recovered CPDs in the anisotropic electric field for all of the compounds are presented in Figures S10 and S11.

For most of the structures, application of an external field drove the CPD toward head-to-head conformations for the z field and head-to-tail conformations for the x field. This predictable and reproducible behavior justified our approach to the orientation process, as stated in the Materials and Methods section. Importantly, the molecules demonstrated varied susceptibility to the electric field, as seen in the changes in CPD upon field application.

**Figure 11:**
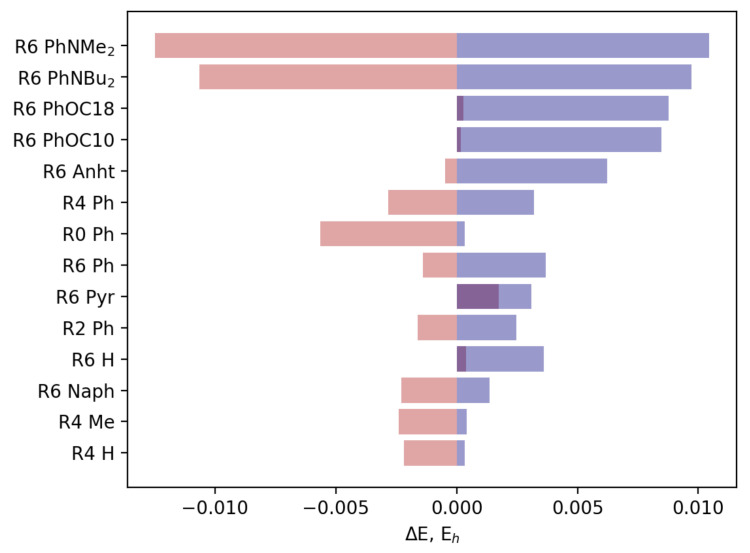
Changes in differences between lowest-lying head-to-head and head-to-tail conformers upon application of an x field (red color) and application of a z field (blue color) relative to original distributions.

For numerical comparison, differences between the lowest-lying head-to-head and lowest-lying head-to-tail conformers in the z and x fields were utilized in Figure 11.

Compounds in series **(III)** demonstrated the highest distribution shifts in both the x and z fields, revealing its high electronic anisotropy and polarizability. A similar significant shift upon the application of a field along the *z* axis was observed for the compounds in series **(IV)**, even though they demonstrated an almost negligible reaction to the application of a field along the *x* axis.

Such calculations deal with the alignment of the cumulative electronic dipole of an aggregate along the z and *x* axes. Since all of the aggregates were aligned in a predefined manner such that the *z* axis was orthogonal to a flat “bottom” of a bunch independent of the inclination of its members, field susceptibility became a separate, unambiguous characteristic, somewhat similar to the polarizability. In the special case of aggregates, this was more useful than the molecular dipole moment of a single molecule, as the former had a synergetic character.

Among the compounds in series **(I)** and **(II)**, there were no obvious trend in the field susceptibility in the CPDs of the aggregates, except for the fact the aggregates of the compounds in **(I)** had very poor field susceptibility in general. The most susceptible aggregates of group **(II)** were those of **R6 Anht**, bearing three conjugated rings as a substituent. Field susceptibility in the group declined in the following order: **R6 Anht** > **R6 Ph** > **R6 Pyr** > **R6 Naph**, leading us to the assumption that the parameter was affected by the cumulative aggregate structural-electronic properties, and no single-molecule considerations could describe this order.

### 3.3. Photophysical Behavior of the Aggregates

#### 3.3.1. Vertical Excitations

The spectra of the aggregates represent the ensemble-averaged Gaussian widened vertical excitation lines (Figures S16 and S17).

**Figure 12:**
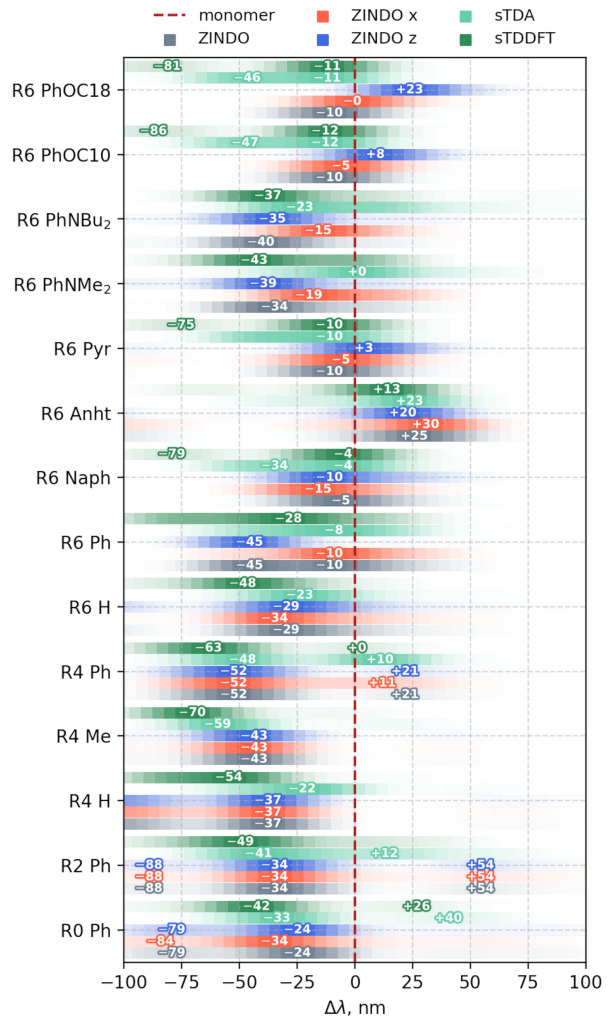
Top view of vertical excitation envelopes for each aggregate aligned to zero according to corresponding monomer maxima. Calculated in different approaches with or without applied anisotropic electric field, numbers indicate peaks with intensities within 10% of the highest peak. Usual form of spectra is presented in Figures S16 and S17.

Since extraction of the exact excitation energies is an overly complicated task for most of the organic chromophores, most attention was paid to the shift in the maxima in the aggregates with respect to the main excitation band of a single molecule at the same level of theory (Figure 12).

In general, it was found that the GFN2-XTB sTDA and sTDDFT calculations imperfectly agreed with each other. Since the main difference between the latter was the de-excitation terms, we could state that their input may be non-negligible in the case of the aggregates.

ZINDO-based spectroscopic results were obtained from a CIS-like calculation with ZINDO/S parametrization. They relatively closely resembled the shifts and band shapes found in the GFN2-XTB sTDA level of theory.

Most pronounced hypsochromic shifts were observed for the aggregates of compounds in series **(I)**, generally in contrast to the susceptibility of their ensembles to application of an external field. Interestingly, not only were the configurational ensembles of these compounds the least susceptible to field application, but also their spectral envelope positions. The peak positions remained mostly intact in the group, pointing out that electronic systems of dense ensembles are generally less polarizable.

The sTDA approach was different from CIS significantly only in this group of compounds, whereas the results of the sTDDFT and CIS calculations on the GFN2-XTB and ZINDO Hamiltonians, respectively, were close in magnitude for their spectral shifts (from monomer to aggregate).

The aggregates of compounds in series **(II)** showed smaller shifts and an opposite tendency to series **(I)**. The GFN2-XTB and sTDA results were closer to those obtained in the ZINDO or CIS approach, suggesting that the proper choice of computational scheme could be very difficult even within the same group of compounds.

The compound **R6 Anht** was an outlier in these calculations, demonstrating a severe batochromic shift barely dependent on the field application. Interestingly enough, according to CPD analysis, all of the compounds in the group demonstrated a head-to-head dominant interaction mode, yet this resulted in a batochromic shift only for one of the compounds, pointing out that more than just the molecular orientation is responsible for a sign of a shift.

The aggregates of compounds in group **(IV)** demonstrated pretty close values of an aggregation-driven hypsochromic shift among the applied methods and significant (varying) batochromic shifts upon z field application. Such behavior is interesting, suggesting that the photophysical properties of these compounds could be controlled. Interestingly, this was exactly the case, as was discussed in the experimental comparison section.

Correspondence of the H- and J-aggregate-associated shifts in Frenkel exciton theory with the shifts obtained in the CIS and TD-DFT approaches was shown in several papers [41,42,57]. Even though the methods could reproduce the results of exciton theory, these spectral shifts were obtained “from the first principles” and thus should not be directly attributed to any implicit physical phenomenon. Thus, here we refrain from attributing shifts in such a manner and treat them as cumulative electronic properties.

The importance of this viewpoint is especially obvious, considering that the aggregates of compounds in series **(II)** were all oriented in a close manner, though only one compound demonstrated a batochromic shift upon transition from monomer excitation to the predicted aggregate ensemble.

This peculiarity suggested further examinations, since transition density analysis (discussed in the next section) was unable to justify the outstanding batochromic shift of **R6 Anht**. At one level of abstraction below, natural transition orbital (NTO) analysis provided solid reasoning for this effect. For **R6 Ph**, **R6 Naph** and **R6 Pyr**, a bright transition is mostly composed of three molecule-localized pairs of orbitals. At the same time, the transition orbitals of R6 Ahnt are far more delocalized (each spanning 2–3 molecules), leading to smaller excitation energies (the NTOs for compounds **R6 Ph**, **R6 Naph**, **R6 Anht** and **R6 Pyr** are presented in Figures S18 and S19). Since both kinds of excitations are non-local in sum, almost indistinguishable transition density patterns were observed. This indicates that even though TD matrix analysis is a powerful tool, it still provides a big picture, so precautions in interpretation must always be taken.

#### 3.3.2. Transition Density Analysis

Even though analysis of most intense transitions or even their envelopes is a tempting holistic approach, the nature of these transitions should also be considered. Analysis of the atom-partitioned transition density revealed that the most pronounced bands were not of exactly the same nature across the compounds. For further ease of perception, analysis and classification, the transition density partition was further expanded to four core-substituent pairs for each compound (Figures S20–S23).

The values of the diagonal matrix elements represent hole–electron interactions within the selected fragments, and they were always large for the 1c, 2c, 3c and 4c elements (standing for 1 core, 2 core, 3 core and 4 core fragments), indicating that BODIPY was a main chromophore unit in the excitation. Interestingly, the molecules with an aromatic moiety in the μ position had the latter also involved in a transition. An intensity of 1–4p (p standing for “peripherial”) matrix elements was low for the compounds with dense stacking (**R2 Ph** and **R4 Ph**), and it was significantly higher for the compounds with labile stacks, approaching its highest limit for compounds **R6 Ph**, **R6 Naph**, **R6 Anht** and **R6 Pyr**, where interactions within the μ substituent were significantly involved in the excitation. It was beneficial that we could see such interaction modes in our calculations, because from there, we could state that the ensemble generation process and electronic property calculation method were also accounting for peculiar off-center interactions. However, the molecule-to-molecule validation approach, which should be used in this case, is questionable, and we decided to outline this caveat and rely on results from the latter section.

The compounds with dense BODIPY core stacking in series **(I)** had significant pairwise off-diagonal elements, indicating effective intermolecular interaction during excitations, and the same behavior was also observed for compound **R6 H** from group **(II)**.

As a general conclusion, it could be speculated that the direct proximity of the BODIPY domains not just alters the significance of a core-core interaction (well known and logical) but also hinders the involvement of the μ substituent in an excitation. In sparse packs, the periphery of one molecule could effectively interact with the chromophoric moiety of another molecule in a stack.

**Figure 13:**
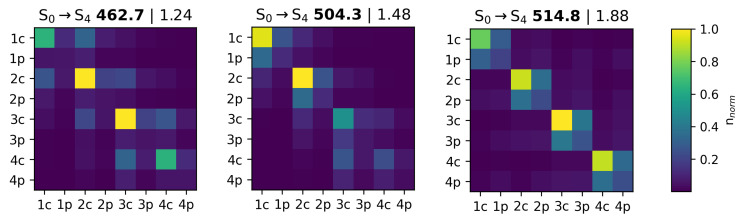
Exemplary fragment-partitioned transition density matrices for (from left to right) **R0 Ph**, **R6 H** and **R6 PhOC18**, demonstrating different intermolecular interaction patterns during the most intense excitations. All of the fragment-partitioned transition density matrices are in Figures S20–S23.

A different degree of core-substituent interaction was responsible for the systematic batochromic shift in the row with the aggregates of group **(II)**, with **R6 H** excluded (because of the lack of aromatic substituent), exceeding the trend in the row of the corresponding BODIPY monomers. Different involvement by the peripherials in the excitation was also observed for the compounds in group **(IV)**, even though the predicted monomer bands were supposed to be shifted batochromically upon switching to a longer chain length, and the opposite trend was observed in the generated aggregates and in the experiment. The same trend was observed in the calculations of the aggregates of the compounds in group **(IV)**. Examples of the transition density matrices can be found in Figure 13.

### 3.4. Experimental Results

The changes observed upon aggregate formation in series **(I)** and for **R6 H** and **R6 Ph** were compared to the results published in Descalzo et al. [20] and were found to qualitatively reproduce the experimental data in a better fashion than expected.

There is a limitation to be spoken of: the experimental results often compare aggregated bands in certain concentrational conditions in solutions. In our calculations, we were dealing with supposedly fully aggregated dyes, so it was hard to reproduce the experimentally observed differences when they were imposed by the *degree* of aggregation. As such, BODIPY **R0 H** has non-negligible water solubility, so it did not aggregate, whereas the calculations suggest it would have possessed both the left and right shoulders if it did. Similarly, differences between **R4 Me** and **R4 H** are mostly imposed by their solubility, which was not obvious from the calculations.

We clearly saw differences between **R4 Me** and **R4 Ph**, with the latter possessing pronounced batochromic bands just as was found in the experiment, since both of those compounds (and all of the others) have poor water solubility. Thus, changes were caused solely by a combination of the organizational and electronic properties of the formed ensembles.

We also observed a similar aggregation effect for **R2 Ph**, but again, it was to almost the same extent as for **R4 Ph**. Qualitatively, they both formed aggregates with batochromic and hypsochromic bands in both the experiments and calculations but at different concentration thresholds.

The calculations also successfully reproduced the differences between **R4 Ph** and **R6 Ph**, showing that the latter only formed hypsochromically shifted aggregate bands, which were responsible for the line broadening.

Quite interestingly, we were also able to reproduce the differences between **R6 H** and **R6 Ph**, showing that the line broadening was larger for the latter due to line splitting in the ensemble. This effect could also describe the lower extinction found for the aggregated **R6 Ph**.

Next, we performed field-oriented calculations to see if we could reproduce the air–water interface effect on the organization and spectroscopic properties. The compounds in series **(IV)** were investigated on an air–water interface in our other study, and it was shown that **R6 PhOC18** formed J-aggregates upon compression on a water subphase.

According to our theoretical investigation, the absorption band of the aggregate of **R6 PhOC18** was batochromically shifted from the absorption band of **R6 PhOC10** to being oriented in an anisotropic electric field applied along the *z* axis. The aggregate bands on the water subphase were also shifted in this manner, though to a smaller extent. The exact strength of the field should be subjected to individual examination, but calibration will only be possible at the point in time when a sufficient amount of spectroscopic data about structures with subphase-driven shifts are available.

In another investigation, we showed that among the variety of BODIPY compounds from group **(II)**, **R6 Anht** showed the most pronounced batochromic shift after deposition onto a glass substrate from the air–water interface [23]. Moreover, we were able to obtain the spectra of highly aggregated films, yet they were barely possible to describe. We found that **R6 Ph** had significant line broadening if completely aggregated. At this point in time, we elaborated that the linear dimensions of the substituent were responsible for this behavior.

**Figure 14:**
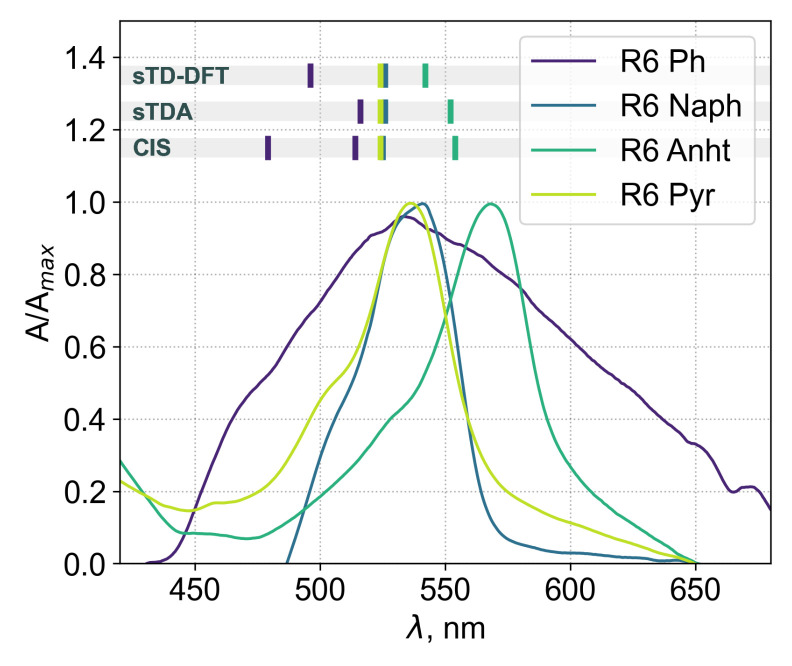
Plot of normalized UV/VIS absorption spectra (solid lines) of aggregated Langmuir–Schaeffer films on a glass substrate of compounds from series **(II)** and maxima shifted according to calculations with respect to hexane solutions.

Figure 14 shows the absorption spectra of the aggregated Langmuir–Schaeffer films, plotted along with dashed lines representing the positions of the aggregate absorption bands calculated according to the shifts from our current results (each line is a hexane maximum with an added calculated shift). Note that two lines are indicated for the CIS calculation of **R6 Ph** because of the broad bimodal peak nature. The band shapes for sTDA and sTD-DFT were also broad compared with the envelopes in the row but had two maxima which were poorly distinguishable. (For a band shape reference, see Figure 12, where the top view of the spectra is presented.)

In this study, we were able to reproduce “from the first principles” the batochromic shift in the aggregated films of **R6 Anht** with exceptional precision and, qualitatively, the broadening of the absorption band for the aggregated film of **R6 Ph**.

Our current results thus were both verified by old experimental data and insightful for their further understanding.

## 4. Conclusions

A new method for the sampling of aggregate configurational spaces allowed us to successfully analyze the representative ensembles of BODIPY aggregates.

We outlined the properties unavailable from single-molecule analysis. The latter included the aggregate ensemble polarizability, electronic excitation band positions in an anisotropic electric field, excitation nature and predominant aggregate geometry. Molecular design considerations should thus be carefully revisited from the perspective of the synergetic effect, which is accessible as a whole from the provided algorithm.

For the investigated group of compounds, we were able to state the following.

Compounds with sparse aliphatic substitutions form dense aggregates with highly distributed transition densities, leading to low polarizability. The ensembles are almost intact upon anisotropic electric field application, and so were their excitation bands. The aggregates for those compounds are generally oriented head to tail and demonstrate a hypsochromic shift in the excitation maxima with respect to the monomers.

Increasing the amount of aliphatic substituents drives the aggregates out of planar stacking, decreasing chromophore interaction upon excitation and resulting in higher susceptibility of the aggregate ensembles to an electric field.

The compounds with conjugated aromatic substituents showed no regular trend in their properties, indicating competing mechanisms in the synergetic aggregate properties. Their aggregates are driven into head-to-head preferred conformations not just due to stacking interactions between the substituents but also due to H⋯F hydrogen bonding interactions.

The introduction of elongated aliphatic substituents could lead to different organizational consequences. Compounds with very long decyloxy- and octadecyloxy-moieties were organized head to head due to the hydrophobic interactions of alkyl chains, whereas differences between the dimethylamino- and dibutylamino-substituted compounds were not that pronounced and, to some extent, demonstrated opposite trends to the former.

Sparse packing also led to the increased involvement of a μ substituent in the electronic excitations, again leading to properties unexplorable with single-molecule examinations.

Ultimately, we were able to state that simplified semi-empirical electronic structure methods provide insightful information about the spectroscopic properties of compounds with our sampling process.

With our available experimental data, we were able to describe the photophysical peculiarities in a row of compounds with varied sizes of aromatic moiety, reproduce the spectral effects of dye aggregation in a water-acetonitrile solution and describe the behavior of **R6 PhOC10** and **R6 PhOC18** in a potential air–water interface. From there, we made the preliminary conclusion of the validity of the presented protocol.

The effectiveness of the applied procedure should definitely be subjected to close-up verification, though the collection of data on dye aggregation is required. We intend to put our further effort in that direction.

## Figures and Tables

**Table 1:** Amount of generated structures in configurational ensembles for each molecule. In the last column and the column captions, HH stands for head-to-head and HT stands for head-to-tail configurations.

Molecule	HH	HT	Total	Lowest
R0 Ph	175	200	375	HT
R2 Ph	158	138	296	HT
R4 Ph	135	122	257	HT
R4 H	219	215	434	HT
R4 Me	214	208	422	HT
R6 H	148	127	275	HT
R6 Ph	62	46	108	HH
R6 Naph	67	14	81	HH
R6 Anht	15	2	17	HH
R6 Pyr	39	10	49	HH
R6 PhNMe${}_{2}$	108	81	189	HT
R6 PhNBu${}_{2}$	77	53	130	HT
R6 PhOC10	110	87	197	HH
R6 PhOC18	108	87	195	HH

## Supplementary Materials

The following supporting information can be downloaded at https://www.mdpi.com/article/10.3390/ijms231810955/s1. Figure S1: ZINDO/CIS, sTDA and sTDDFT Spectral envelopes yielded by two-step generation process of BODIPY aggregate with 3 molecules, Figure S2: ZINDO/CIS, sTDA and sTDDFT Spectral envelopes yielded by two-step generation process of BODIPY aggregate with 4 molecules, Figure S3: ZINDO/CIS, sTDA and sTDDFT Spectral envelopes yielded by two-step generation process of BODIPY aggregate with 5 molecules, Figure S4: ZINDO/CIS, sTDA and sTDDFT Spectral envelopes yielded by two-step generation process of BODIPY aggregate with 6 molecules, Figure S5: ZINDO/CIS, sTDA and sTDDFT Spectral envelopes yielded by one-step generation process of BODIPY aggregate with 4 molecules, Figure S6: ZINDO/CIS, sTDA and sTDDFT Spectral envelopes yielded by three-step generation process of BODIPY aggregate with 4 molecules, Figure S7: Configurational probability distribution histograms for ensembles of studied compounds, Figure S8: Lowest energy aggregate structures for compounds from groups (I) and (II), Figure S9: Lowest energy aggregate structures for compounds from groups (III) and (IV), Figure S10: Configurational probability distribution histograms for ensembles of studied compounds with applied anisotropic electric field (along *x*-axis), Figure S11: Configurational probability distribution histograms for ensembles of studied compounds with applied anisotropic electric field (along *z*-axis), Figure S12–S15: Hirschfield fingerprints for two inside molecules of a tetramer (a) and (b) and analysis of atom pair input (histograms on the right), Figure S16: ZINDO/S envelopes of aggregate ensembles with or without applied anisotropic electric field (solid lines) and corresponding monomeric molecules (dashed line), Figure S17: sTDA and sTDDFT envelopes of aggregate ensembles (solid lines) and corresponding monomeric molecules (dashed lines), Figure S18: Natural transition orbitals of compounds R6 Ph (top) and R6 Naph (bottom) involved in a brightest excitation, numbers between orbitals are occupation numbers, Figure S19: Natural transition orbitals of compounds R6 Anht (top) and R6 Pyr (bottom) involved in a brightest excitation, numbers between orbitals are occupation numbers. Figure S20–S23: Fragment-partitioned transition density matrices for five lowest transitions of corresponding molecules calculated in ZINDO/CIS level of theory. Numbers 1–4 indicate molecule in aggregate, “c” index stands for BODIPY core and “p” index stands for substituents. References [31,33,34,35,36,37,38,40,47,58] are cited in the supplementary materials.
