# Supplementary material for: Semi-Empirical Calculation of Bodipy Aggregate Spectroscopic Properties through Direct Sampling of Configurational Ensembles
Source: Int J Mol Sci. 2022 Sep 19;23(18):10955. doi: 10.3390/ijms231810955 (PMC9502801; doi:10.3390/ijms231810955)
Supplement: Supplementary file 1 [file ijms-23-10955-s001.zip › ijms-1905044 - Supplementary Material.pdf]

SEMI-EMPIRICAL CALCULATION OF BODIPY AGGREGATE SPECTROSCOPIC PROPERTIES THROUGH DIRECT SAMPLING OF CONFIGURATIONAL ENSEMBLES

September 19, 2022

Supplementary information

S1 Comparison of computational methods

Electronic structure methods

All of the electronic structure methods utilized in the research are discussed in a broad details elsewhere as it's going to be mentioned in references. Even though, short recap is provided below.

Vertical excitation energies in TD-DFT problem formulated from Kohn-Sham (or Hartree-Fock) equation after applied oscillatory perturbation are eigenvalues ω of Casida equations [58](Equation S1):

$$\begin{pmatrix} A & B \\ B^* & A^* \end{pmatrix} \begin{pmatrix} X \\ Y \end{pmatrix} = \omega \begin{pmatrix} 1 & 0 \\ 0 & -1 \end{pmatrix} \begin{pmatrix} X \\ Y \end{pmatrix} \tag{1}$$

Where matrices A and B contain one-electron energy differences ϵ, two-electron integrals for virtual-occupied and occupied-virtual interactions, respectively, as well as exchange-correlation integrals, both of the latter scaled by the amount of semi-local density functional exchange a_x. The X and Y are matrices containing unknown interaction amplitudes (CIS amplitudes) of transitions between occupied and virtual orbitals (Equation S2) [31].

$$\begin{aligned} A_{ia,jb} &= \delta_{ij}\delta_{ab}(\epsilon_a - \epsilon_i) + 2(ia|jb) - a_x(ij|ab) + (1 - a_x)(ia|f_{xc}|jb) \\ B_{ia,jb} &= 2(ia|bj) - a_x(ib|aj) + (1 - a_x)(ia|f_{xc}|bj) \end{aligned} \tag{2}$$

In Tamm-Danchoff approximation, de-excitation terms are neglected, yielding form (Equation S3) of an eigenvalue problem, being twice less computationally

demanding than original form (Equation S1):

$$AX = \omega X \tag{3}$$

Simplifications introduced by Grimme and co-workers include: neglect of exchange-correlation response (time-independent approximation), substitution of the two-electron integrals with a damped Coloumb interactions of charge/density monopoles and finally, restriction of the configuration interaction space to a reasonable, user-defined range [35-36].

Semi-empirical quantum chemistry methods

GFN2-XTB and Zerner's INDO approaches deal with the Roothan-Hall eigenvalue problem of the form (Equation S4):

$$FC = SC\epsilon \tag{4}$$

Where F is a Fock matrix, S is an overlap matrix, C is an atomic orbital coefficients matrix and ϵ is a matrix of orbital energies.

GFN2-XTB, utilizes a minimal valence basis set of atom centered, contracted Gaussian functions, which approximate Slater functions (STO-mG) with additional polarization functions for heavy (Z>9) main group elements, and the Fock matrix reads (Equation S5):

$$F_{k\lambda} = H_{k\lambda} + F_{k\lambda}^{IES+IXC} + F_{k\lambda}^{AES} + F_{k\lambda}^{AXC} + F_{k\lambda}^{D4} \tag{5}$$

Where H is an extended Hueckel matrix, indices IES, IXC denote anisotropic electrostatic and exchange-correlation contributions, treated as shell-partitioned Mulliken charges with interactions clamped according to Mataga-Nishimoto-Ohno-Klopman equation.

Anisotropic electrostatic and exchange-correlation interaction terms AES and AXC are extensively parametrized second-order expansions of multipole interaction energies intended to account for effects of charge distribution anisotropy (to better describe a non-covalent interactions, allowing to throw off H-bond and halogen bond corrections) and compensate for a small basis set (partially compensating for a polarization function absence at small atoms), respectively. The last term, $D4$, stands for a self-consistent version of a London dispersion correction [33-34,47].

Intermediate neglect of differential diatomic overlap, though complicated for direct comparison, also described in brief below. Method utilizes minimal valence basis set and has the following form of a Fock matrix (Equa-

tion S6):

$$\begin{aligned} F_{\mu\mu} &= U_{\mu\mu} + \sum_{B\neq A} V_{\mu\mu,B} + \sum_{\nu\in A} P_{\nu\nu}(\gamma_{\mu\nu} - \frac{1}{2}\eta_{\mu\nu}) \\ &\quad + \sum_{B\neq A}\sum_{\lambda\in B} P_{\lambda\lambda}(\mu\mu|\lambda\lambda), (\mu,\nu \in A, \lambda \in B) \\ F_{\mu\nu} &= \frac{1}{2}P_{\mu\nu}(3\eta_{\mu\nu} - \gamma_{\mu\nu}), (\mu,\nu \in A) \\ F_{\mu\lambda} &= \beta_{\mu\lambda} - \frac{1}{2}P_{\mu\lambda}(\mu\mu|\lambda\lambda), (\mu \in A, \lambda \in B) \end{aligned} \tag{6}$$

The indices μ and ν are corresponding to atomic orbitals of atom A, index λ is corresponding to atom B ($B \neq A$), $U_{\mu\mu}$ is the one-center one-electron energy, $\gamma_{\mu\nu}$ and $\eta_{\mu\nu}$ are one-center Coloumb and exchange integrals, respectively (parametrized form experimental atomic spectra).

$V_{\mu\mu}$ is a core-electron attraction term, $\beta_{\mu\nu}$ is a resonance integral comprising purely empirical terms and an overlap matrix, and finally, $(\mu\mu|\lambda\lambda)$ are two-center Coloumb integrals.

Zerner's INDO approach suggests only calculation of the two-center two-electron integrals of a kind $(\mu\mu|\lambda\lambda)$, explicitly assumes they are independent of the form of atomic orbitals and are calculated via Mataga-Nishimoto formula. One-center two-electron integrals are parametrized from a Pariser approximation to be a difference between the corresponding electron affinities and ionisation potentials [37-38,40].

Both of the computational methods bear known problems of a minimal Slater-type orbital basis set and Hartree-Fock method itself, yet as described above, GFN2-XTB method overcomes limitations due to introduction of polarization functions and careful treatment of anisotropic electronic interactions, responsible for the weak interactions holding together molecular aggregates. Due to the benefits, GFN2-XTB was utilized in this research for geometry optimization, single point energy evaluations and as a base for sTDA and sTDDFT calculations as implemented in XTB, STDA, orca and DFTB+ packages.

ZINDO is, in turn, lacking any electron correlation and bears too crude of an integral approximation, and thus does not include any nonbonding interactions, rendering method barely sufficient for geometry optimizations, and absolutely infeasible for molecular aggregate geometry analysis.

Even though, due to the involved parametrization, ZINDO/S (S stands for "Spectroscopic") method does a great job of description of vertical electronic excitations in a configuration interaction singles approach, and was thus utilized in our research to assess aggregate spectroscopic properties from one additional perspective.

S2 Preliminary assessment data

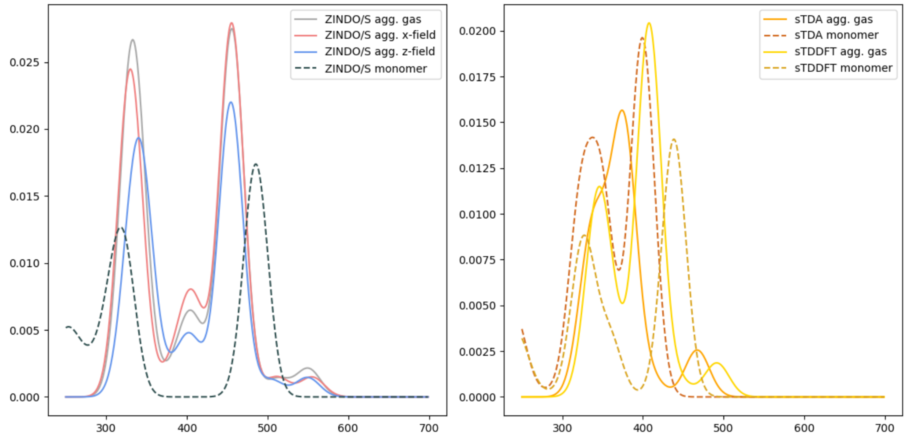

Figure S1: ZINDO/CIS, sTDA and sTDDFT Spectral envelopes yielded by **two-step** generation process of BODIPY aggregate with **3 molecules**

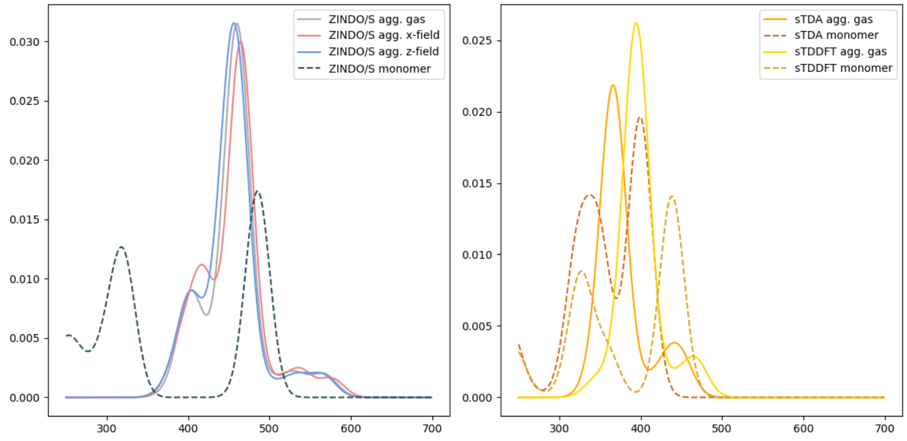

Figure S2: ZINDO/CIS, sTDA and sTDDFT Spectral envelopes yielded by **two-step** generation process of BODIPY aggregate with **4 molecules**

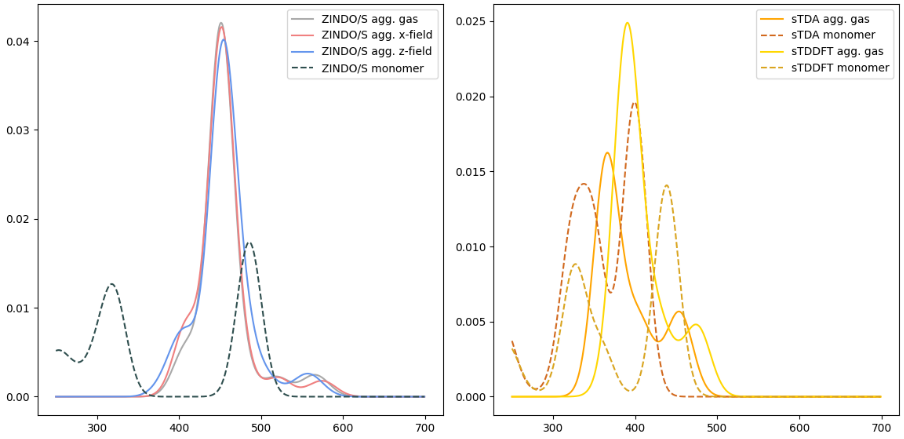

Figure S3: ZINDO/CIS, sTDA and sTDDFT Spectral envelopes yielded by two-step generation process of BODIPY aggregate with **5 molecules**

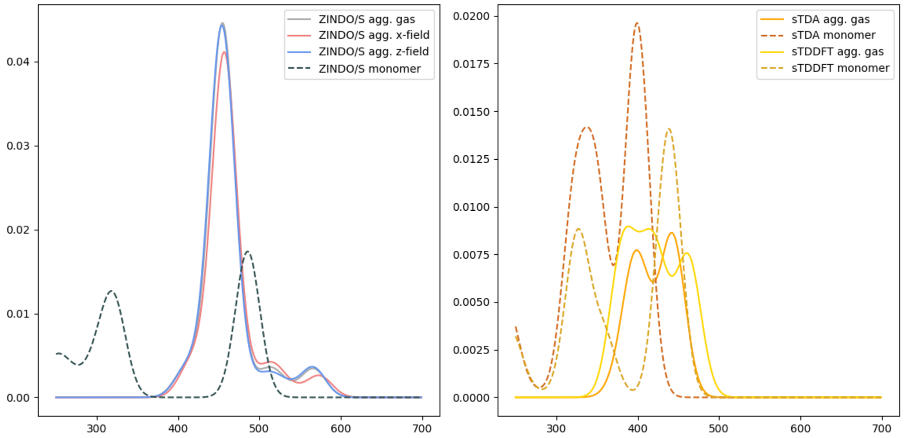

Figure S4: ZINDO/CIS, sTDA and sTDDFT Spectral envelopes yielded by two-step generation process of BODIPY aggregate with **6 molecules**

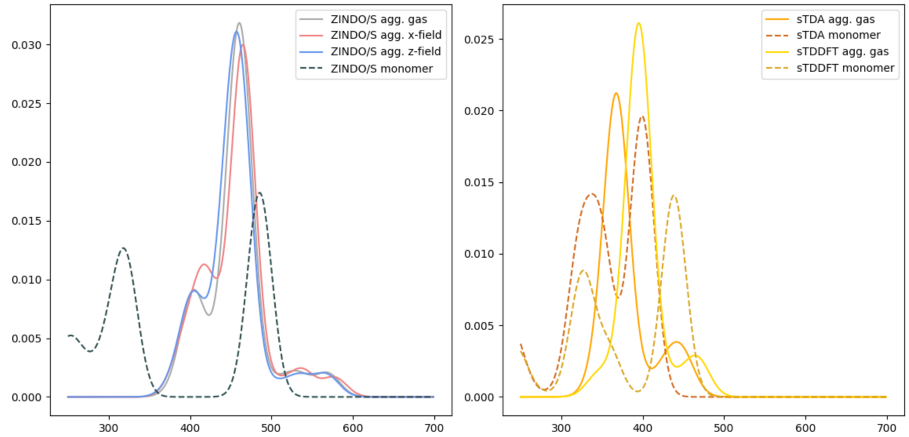

Figure S5: ZINDO/CIS, sTDA and sTDDFT Spectral envelopes yielded by **one-step** generation process of BODIPY aggregate with 4 molecules

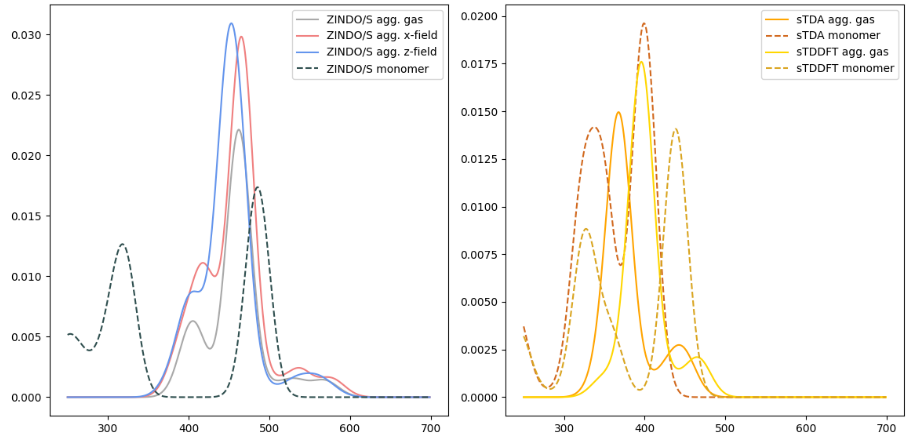

Figure S6: ZINDO/CIS, sTDA and sTDDFT Spectral envelopes yielded by **three-step** generation process of BODIPY aggregate with 4 molecules

S3 Comparative analysis

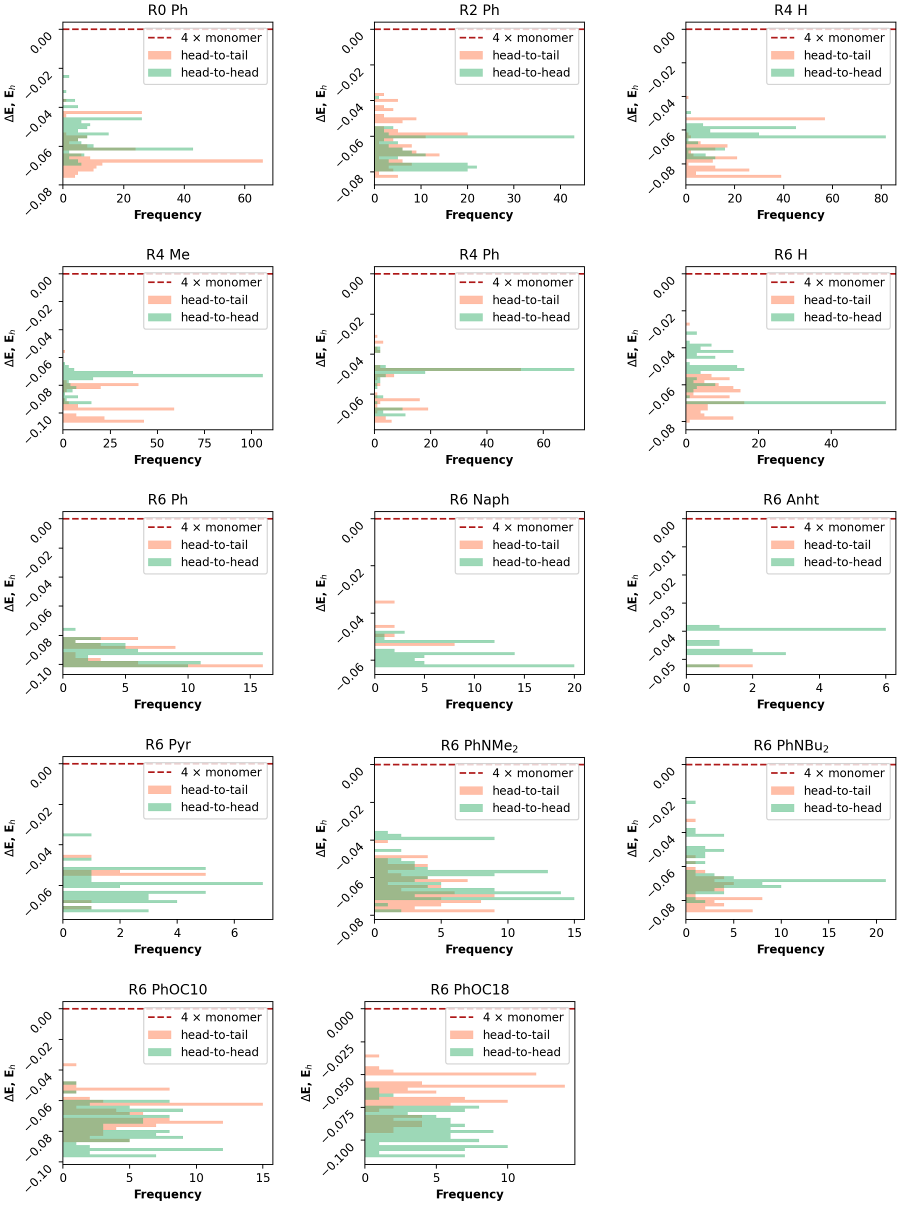

Figure S7: Configurational probability distribution histograms for ensembles of studied compounds

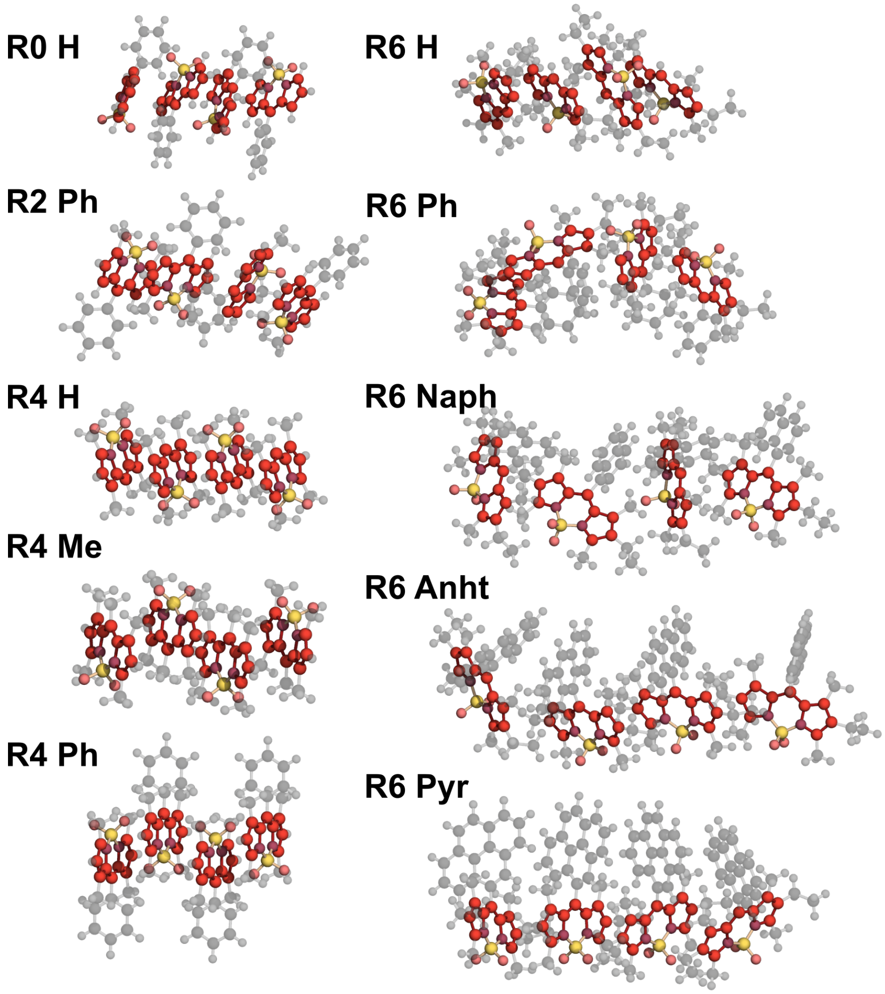

Figure S8: Lowest energy aggregate structures for compounds from groups **(I)** and **(II)**

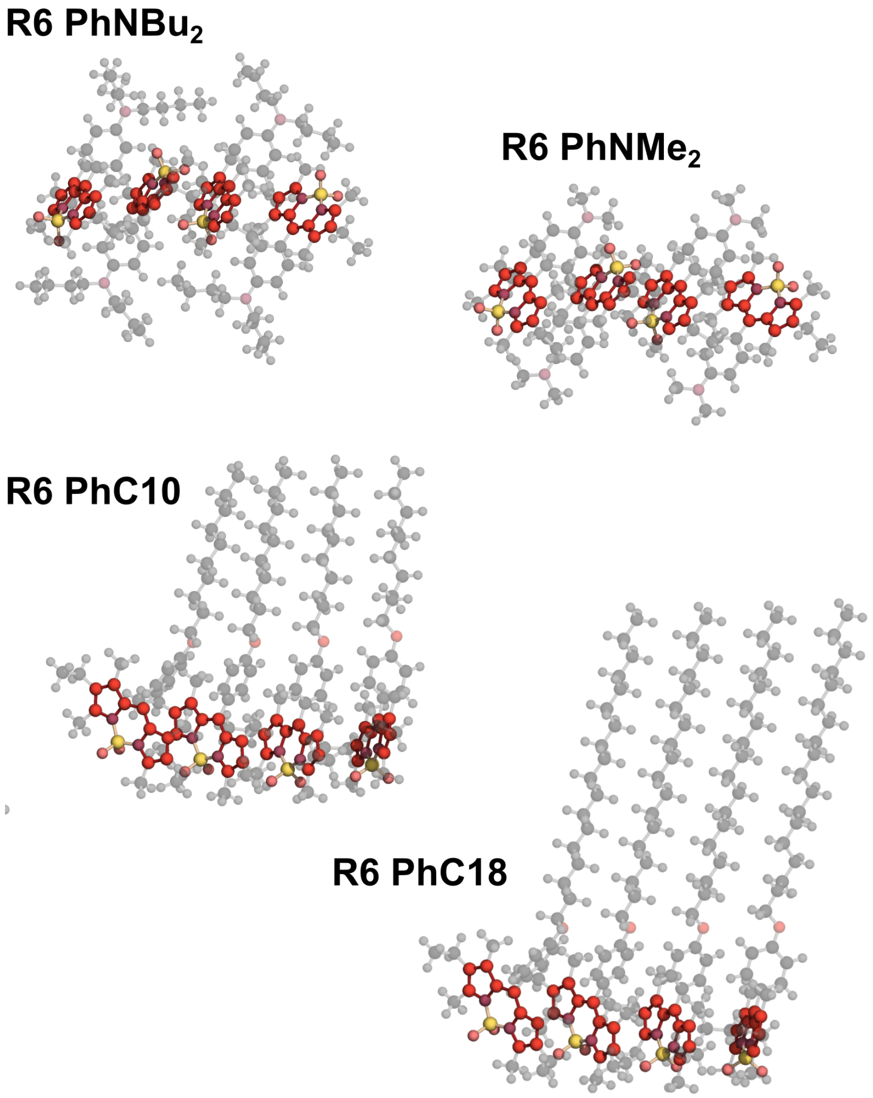

Figure S9: Lowest energy aggregate structures for compounds from groups **(III)** and **(IV)**

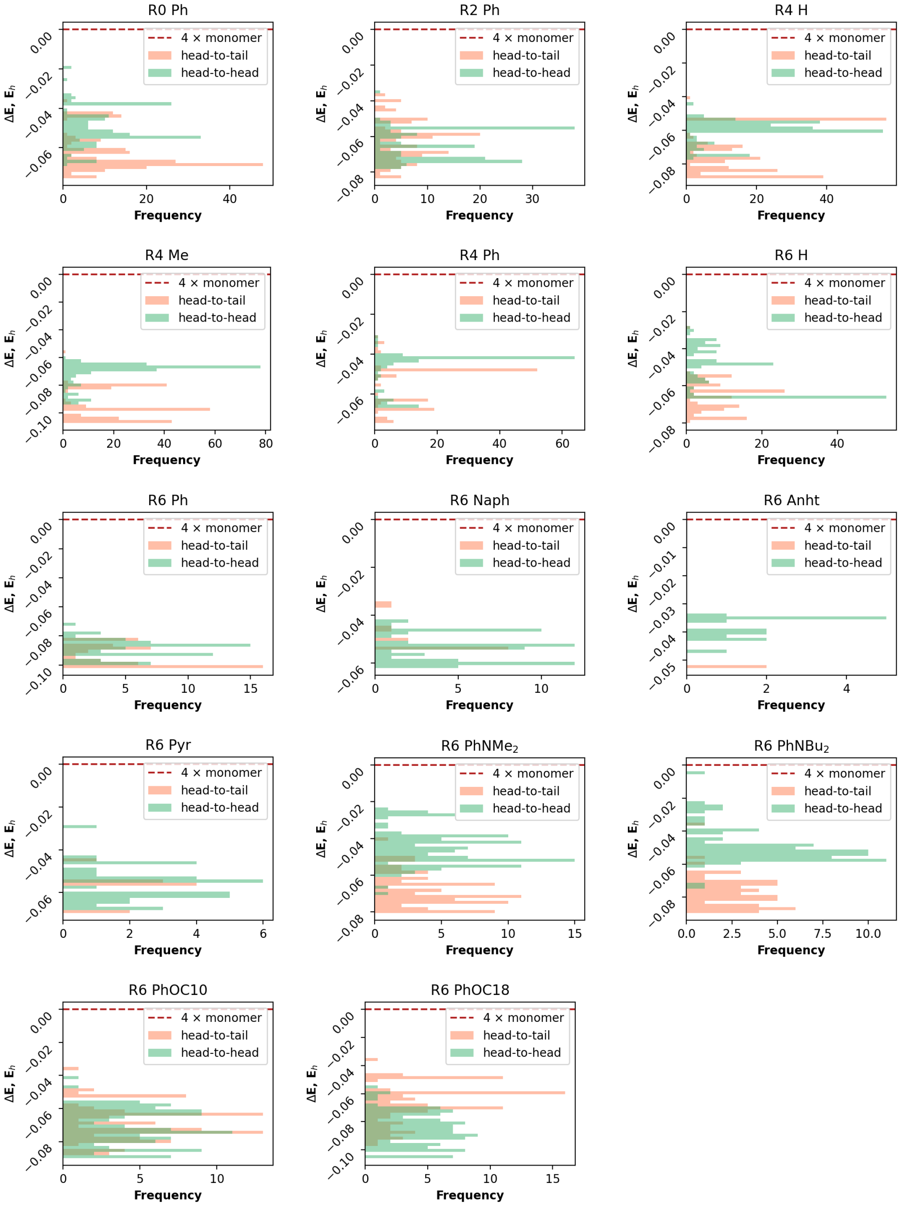

Figure S10: Configurational probability distribution histograms for ensembles of studied compounds with applied anisotropic electric field (along x-axis)

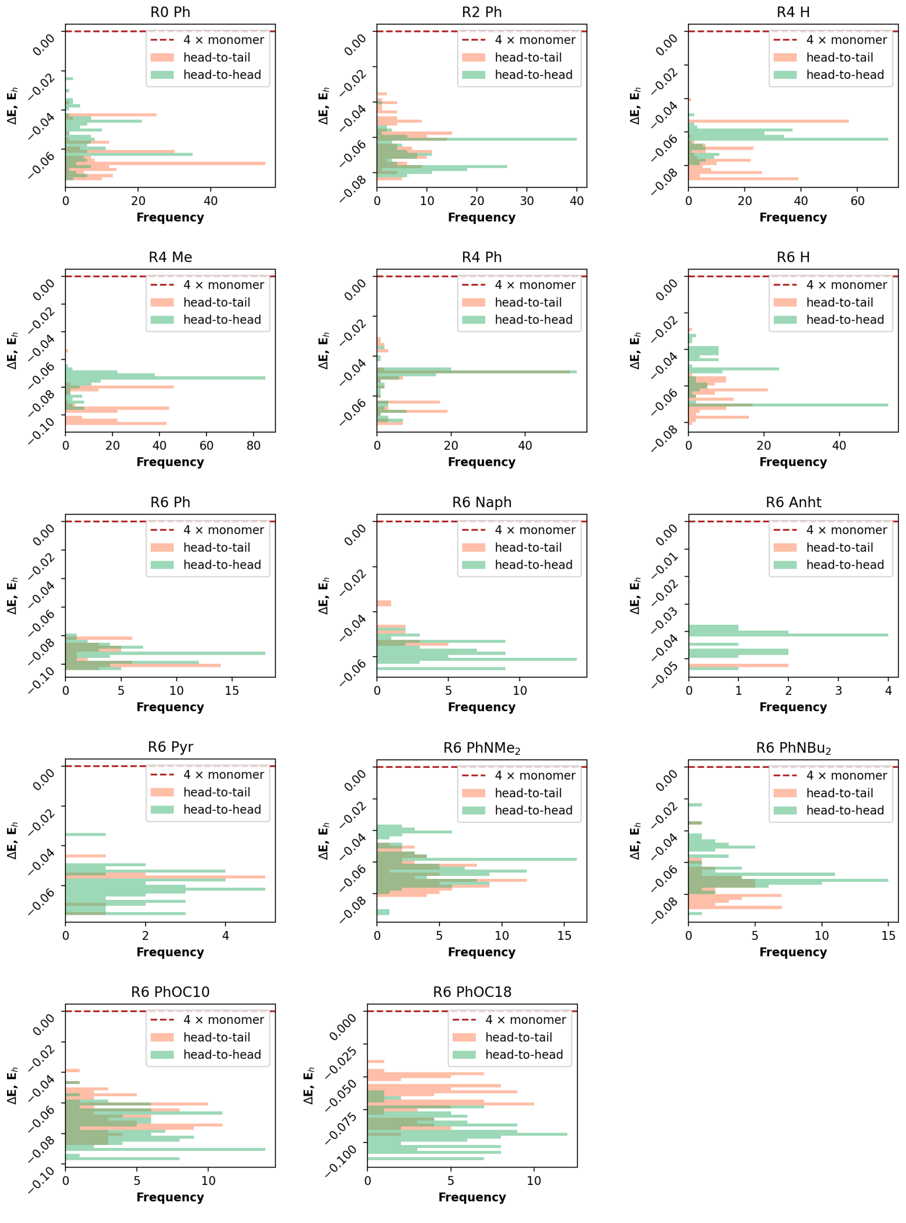

Figure S11: Configurational probability distribution histograms for ensembles of studied compounds with applied anisotropic electric field (along z-axis)

S4 Hirschfield fingerprints

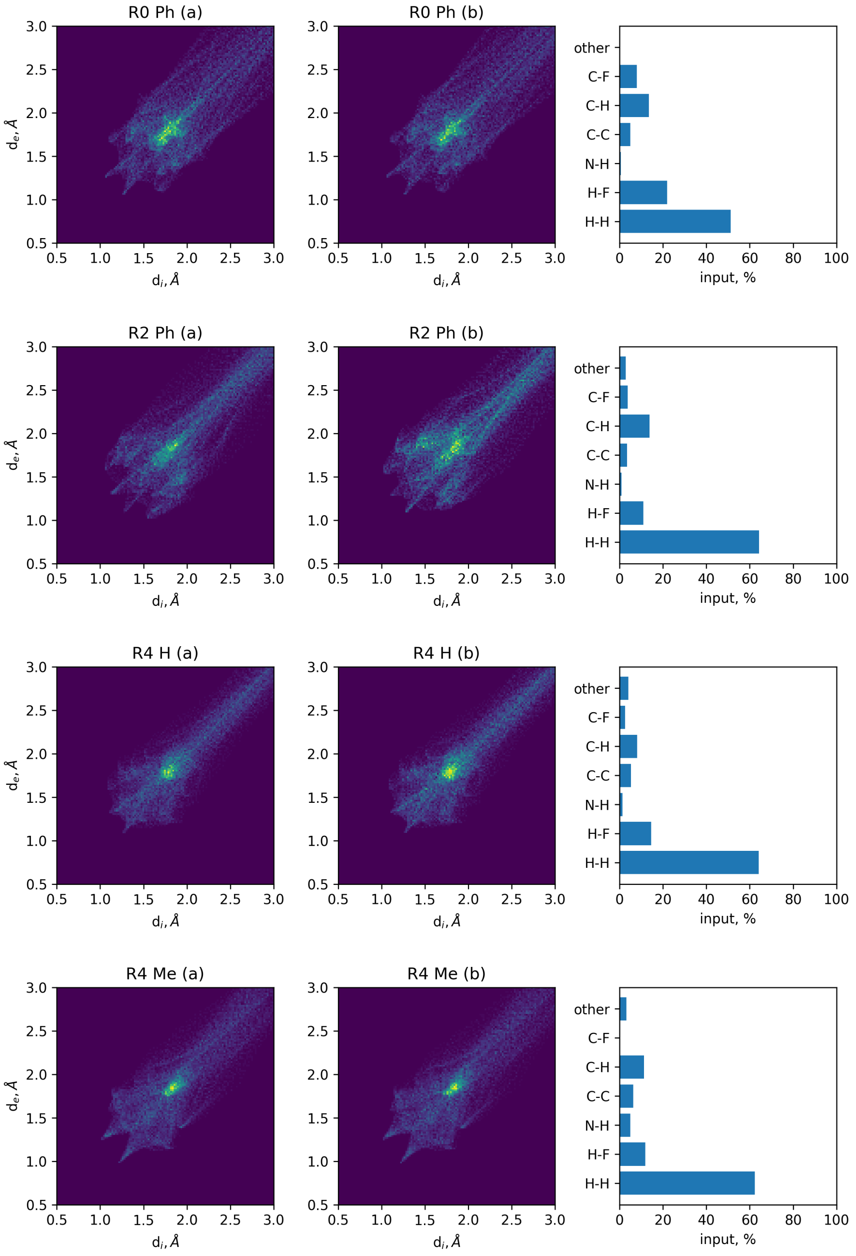

Figure S12: Hirschfield fingerprints for two inside molecules of a tetramer (a) and (b) and analysis of atom pair input (histograms on the right)

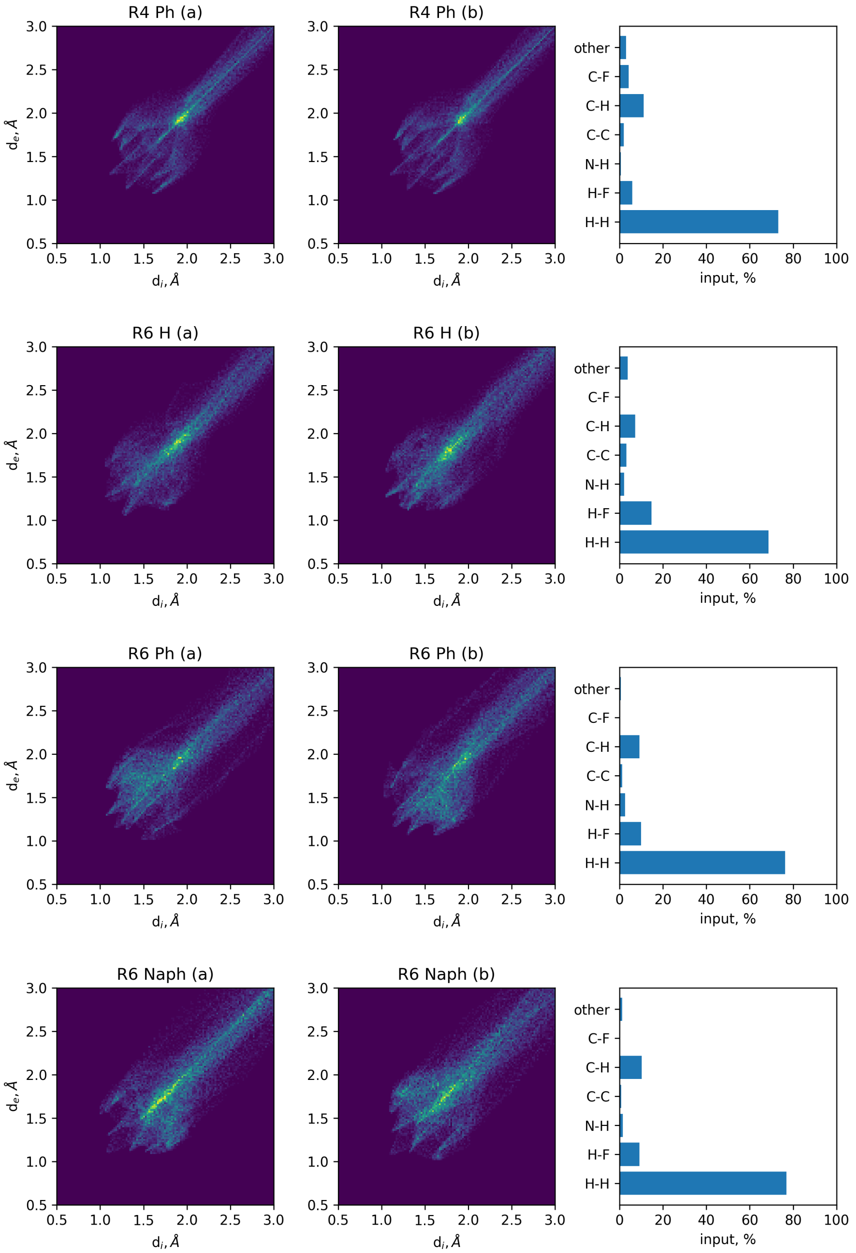

Figure S13: Hirschfield fingerprints for two inside molecules of a tetramer (a) and (b) and analysis of atom pair input (histograms on the right)

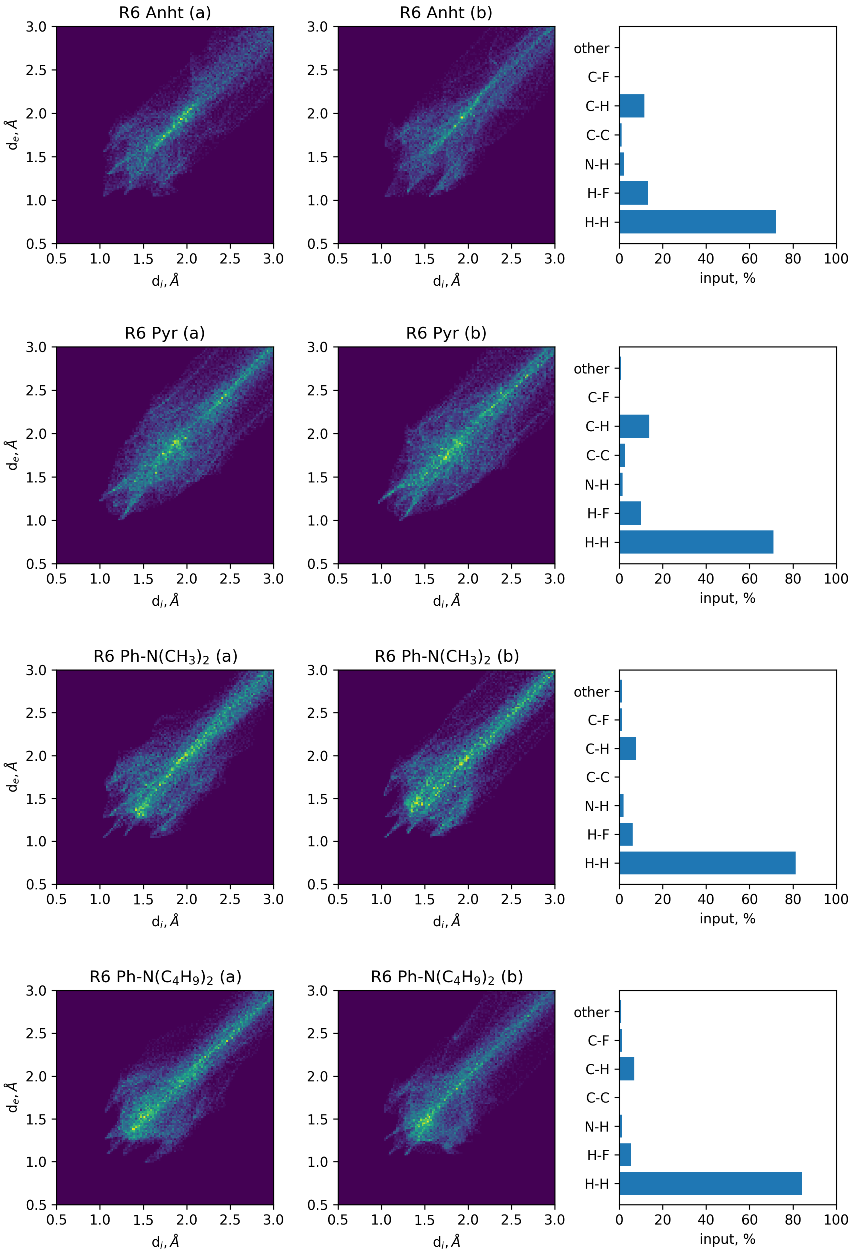

Figure S14: Hirschfield fingerprints for two inside molecules of a tetramer (a) and (b) and analysis of atom pair input (histograms on the right)

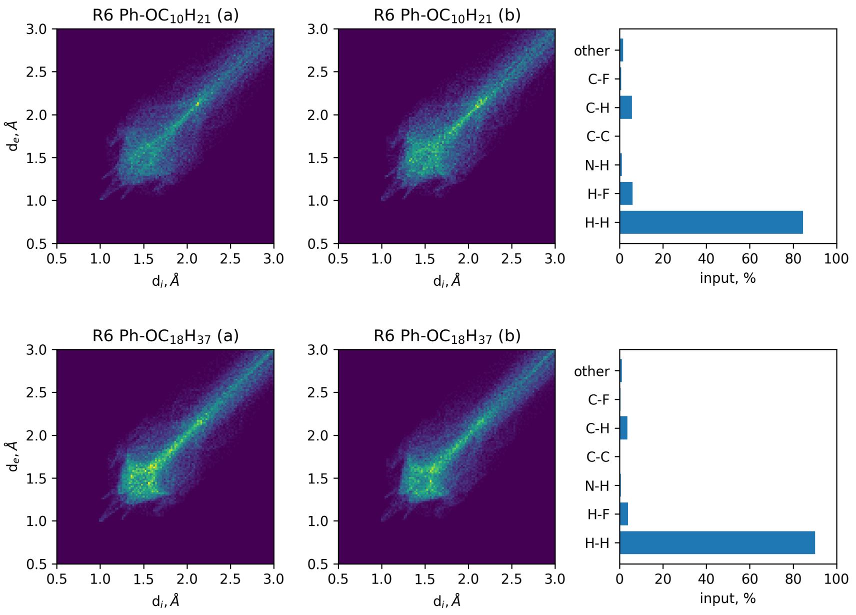

Figure S15: Hirschfield fingerprints for two inside molecules of a tetramer (a) and (b) and analysis of atom pair input (histograms on the right)

S5 Electronic excitation envelopes

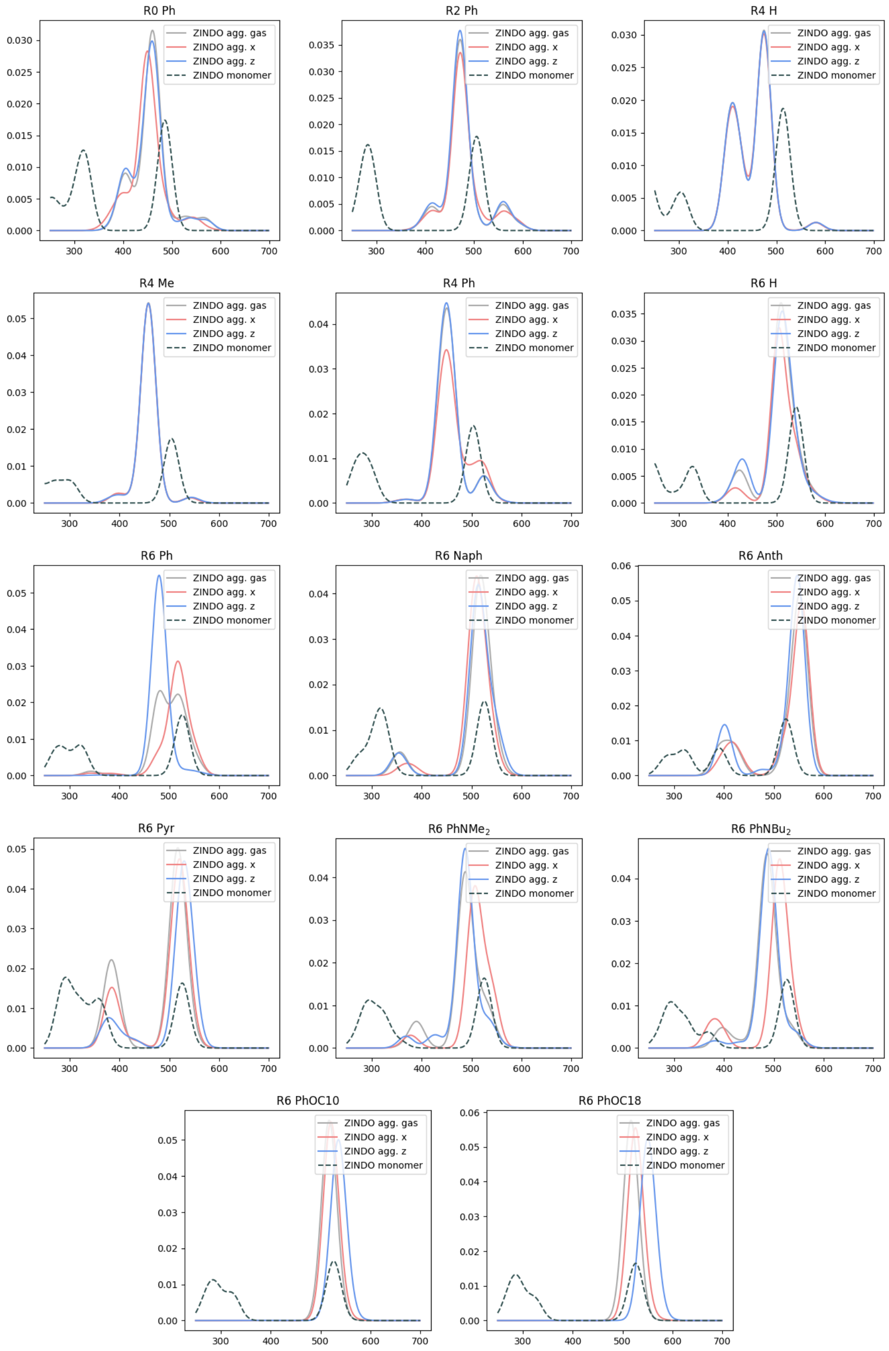

Figure S16: ZINDO/S envelopes of aggregate ensembles with or without applied anisotropic electric field (solid lines) and corresponding monomeric molecules (dashed line)

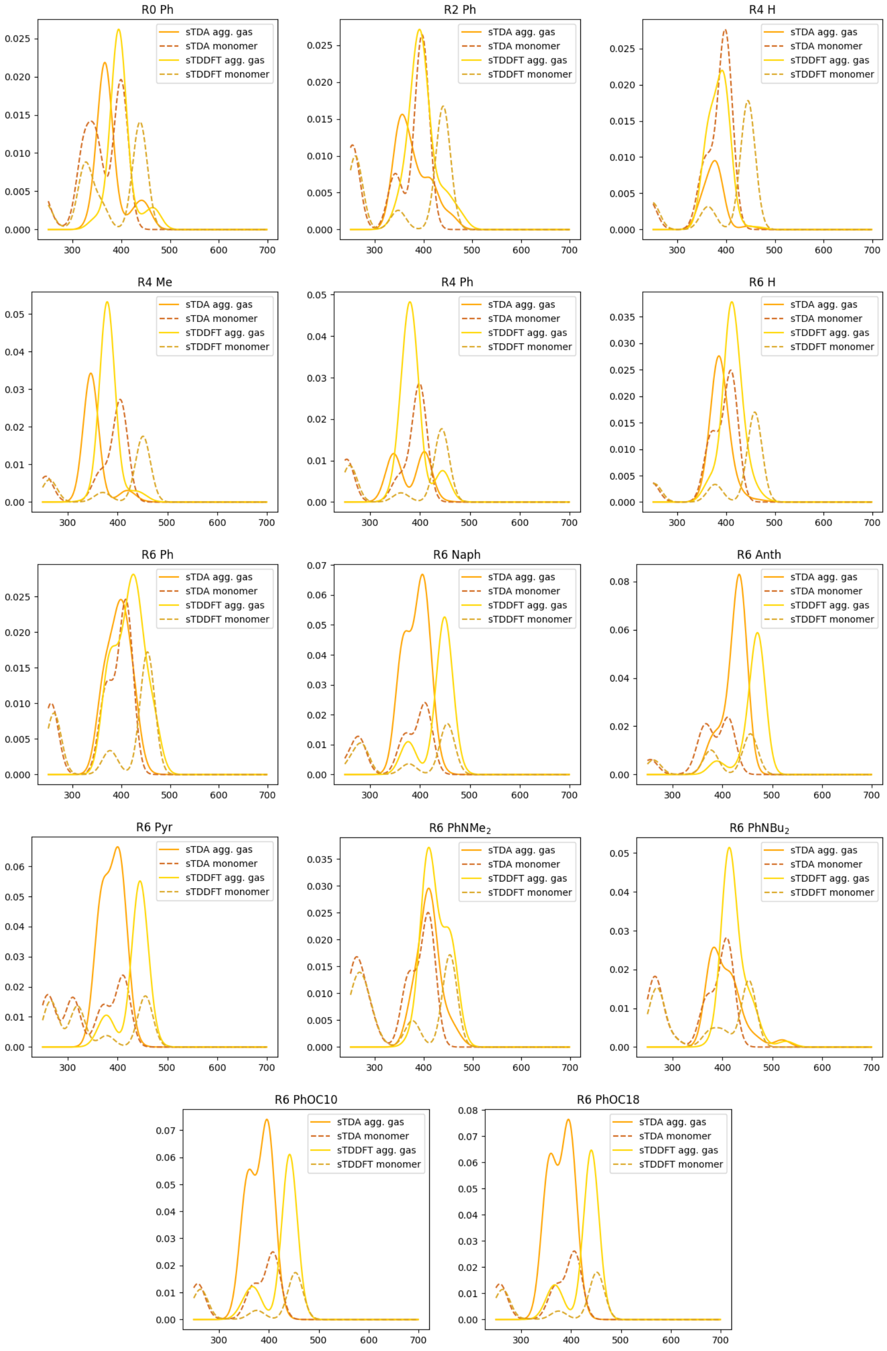

Figure S17: sTDA and sTDDFT envelopes of aggregate ensembles (solid lines) and corresponding monomeric molecules (dashed lines)

S6 Natural transition orbital analysis

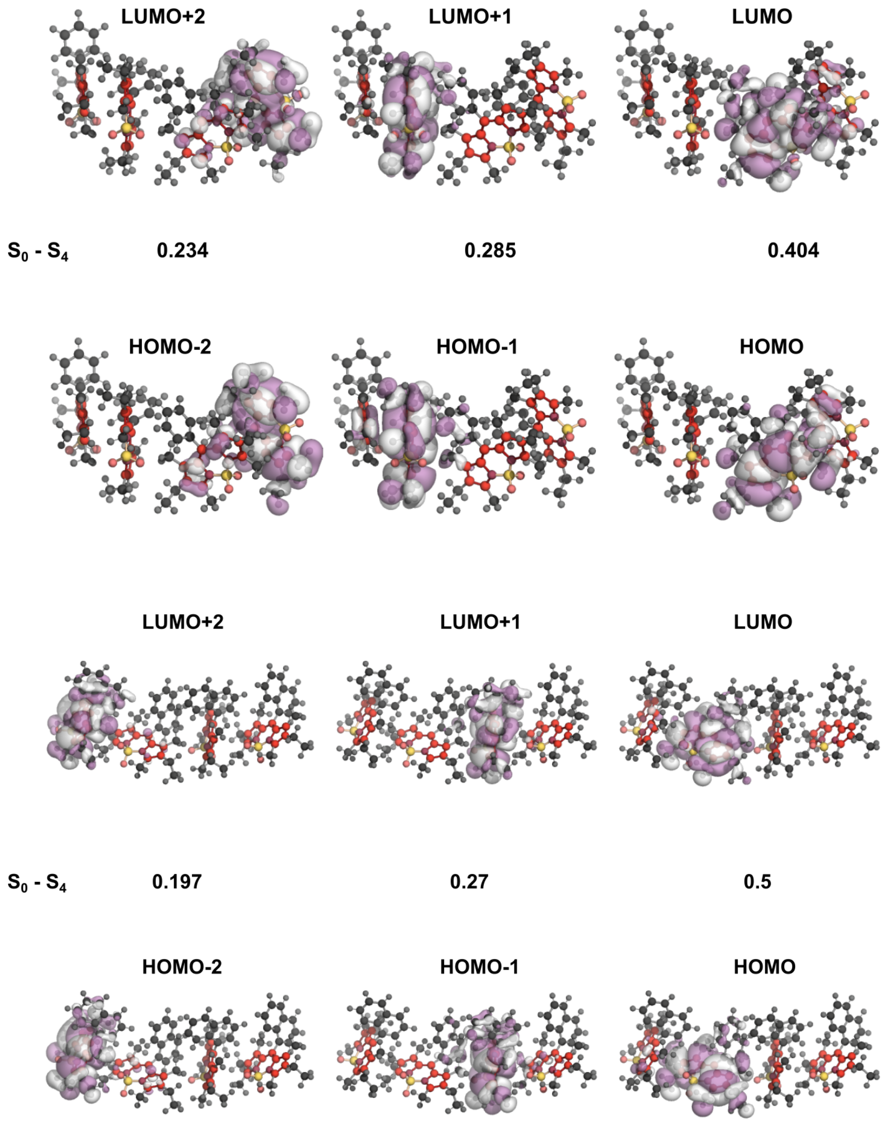

Figure S18: Natural transition orbitals of compounds **R6 Ph** (top) and **R6 Naph** (bottom) involved in a brightest excitation, numbers between orbitals are occupation numbers

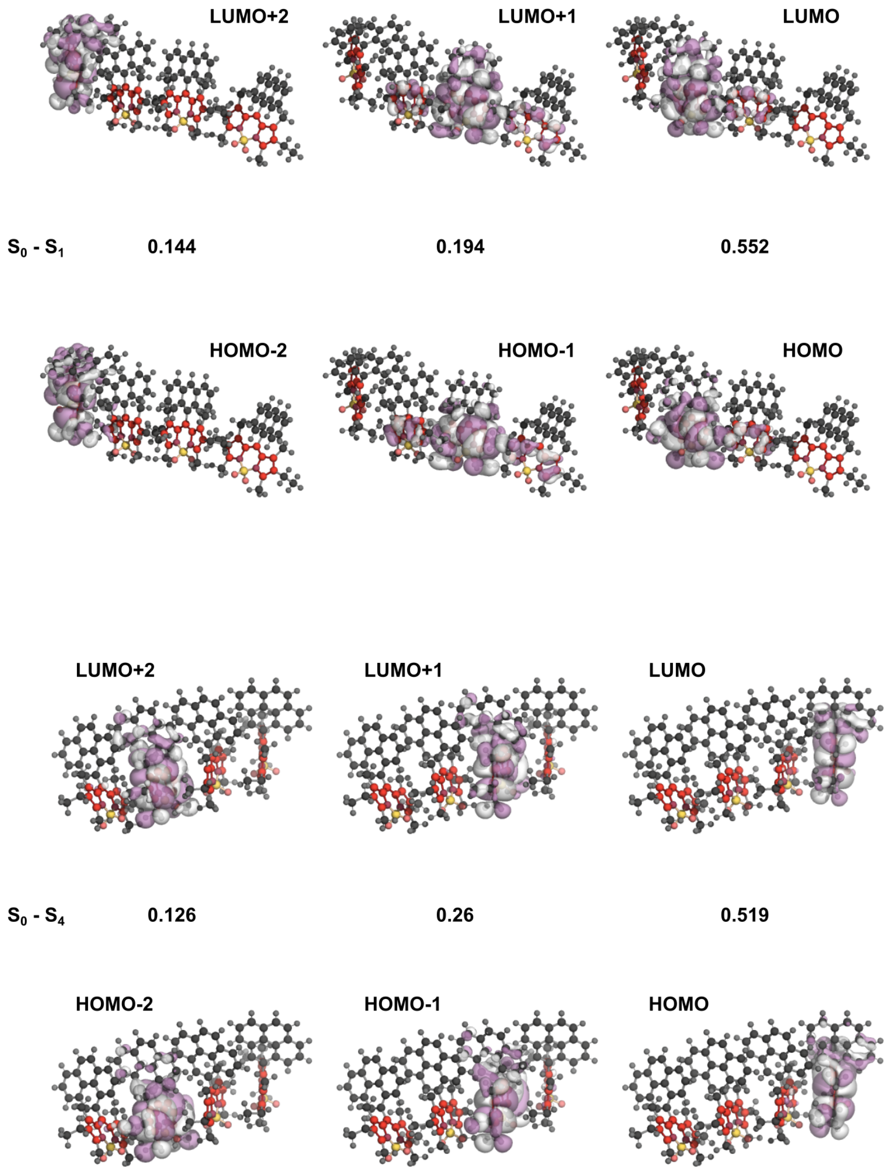

Figure S19: Natural transition orbitals of compounds **R6 Anht** (top) and **R6 Pyr** (bottom) involved in a brightest excitation, numbers between orbitals are occupation numbers

S7 Transition density matrices

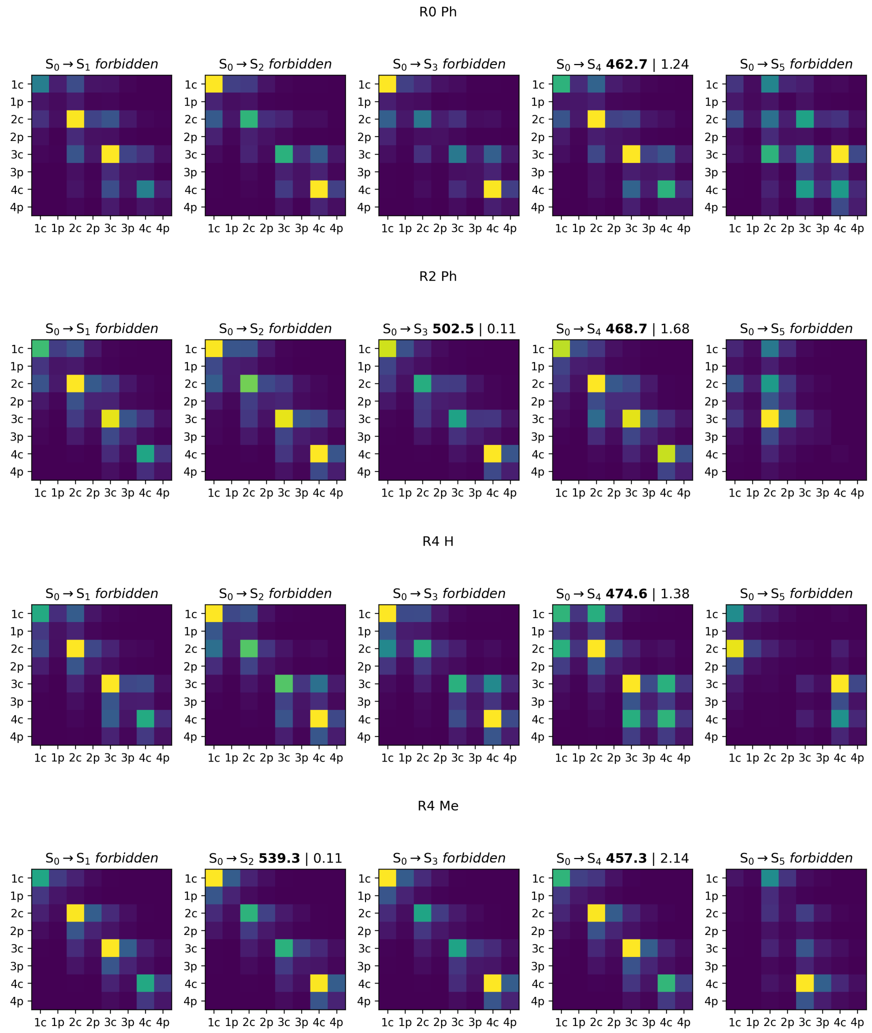

Figure S20: Fragment-partitioned transition density matrices for five lowest transitions of corresponding molecules calculated in ZINDO/CIS level of theory. Numbers 1-4 indicate molecule in aggregate, ”c” index stands for BODIPY core and ”p” index stands for substituents.

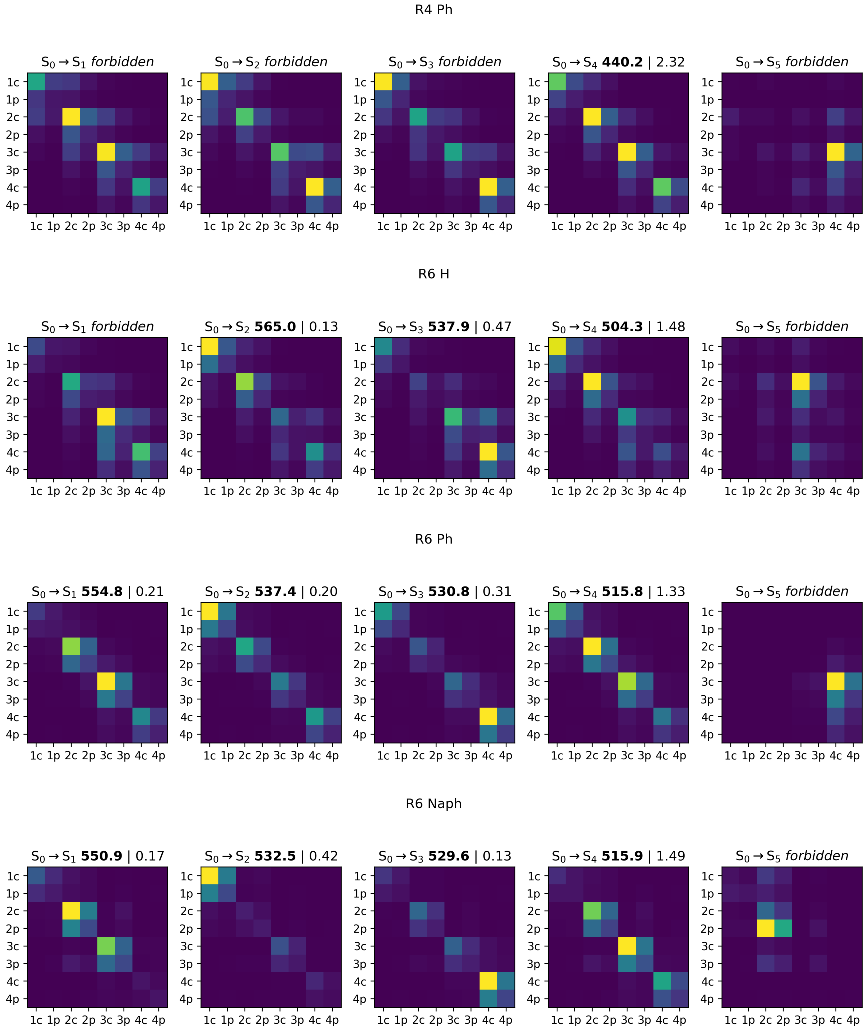

Figure S21: Fragment-partitioned transition density matrices for five lowest transitions of corresponding molecules calculated in ZINDO/CIS level of theory. Numbers 1-4 indicate molecule in aggregate, ”c” index stands for BODIPY core and ”p” index stands for substituents.

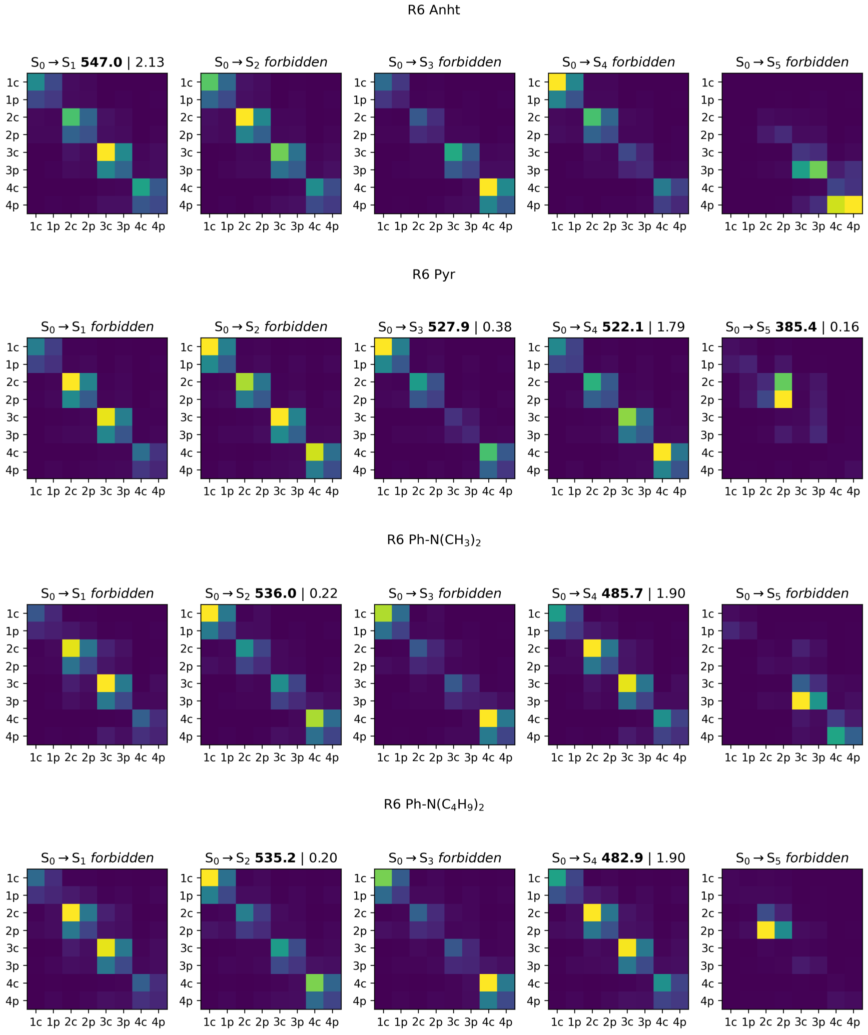

Figure S22: Fragment-partitioned transition density matrices for five lowest transitions of corresponding molecules calculated in ZINDO/CIS level of theory. Numbers 1-4 indicate molecule in aggregate, "c" index stands for BODIPY core and "p" index stands for substituents.

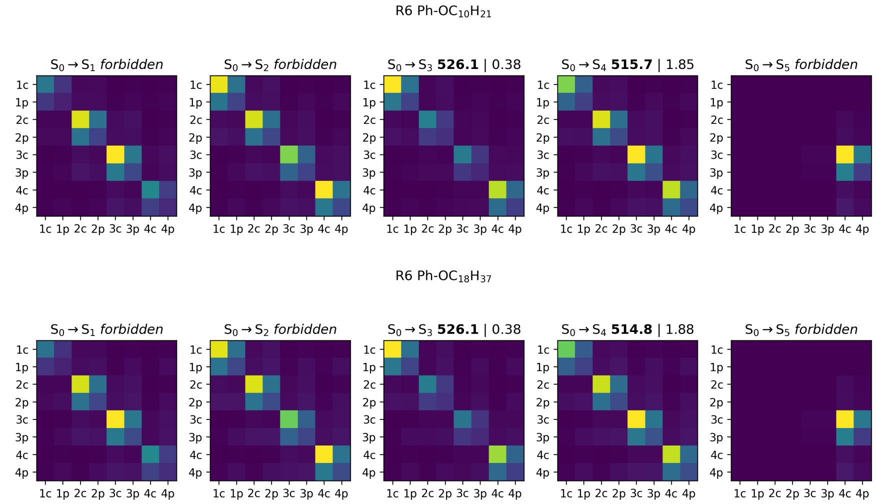

Figure S23: Fragment-partitioned transition density matrices for five lowest transitions of corresponding molecules calculated in ZINDO/CIS level of theory. Numbers 1-4 indicate molecule in aggregate, "c" index stands for BODIPY core and "p" index stands for substituents.
